# Local acting Sticky-trap inhibits vascular endothelial growth factor dependent pathological angiogenesis in the eye

**DOI:** 10.1002/emmm.201303708

**Published:** 2014-04-04

**Authors:** Iacovos P Michael, Peter D Westenskow, Sabiha Hacibekiroglu, Alissa Cohen Greenwald, Brian G Ballios, Toshihide Kurihara, Zhijie Li, Carmen M Warren, Puzheng Zhang, Edith Aguilar, Laura Donaldson, Valentina Marchetti, Takeshi Baba, Samer M Hussein, Hoon-Ki Sung, M Luisa Iruela-Arispe, James M Rini, Derek van der Kooy, Martin Friedlander, Andras Nagy

**Affiliations:** 1 Lunenfeld-Tanenbaum Research Institute, Mount Sinai HospitalToronto, ON, Canada; 2 Department of Cell and Molecular Biology, The Scripps Research InstituteLa Jolla, CA, USA; 3 Institute of Medical Science, University of TorontoToronto, ON, Canada; 4 Department of Biochemistry, University of TorontoToronto, ON, Canada; 5 Department of Molecular, Cell and Developmental Biology, University of CaliforniaLos Angeles, CA, USA; 6 Department of Molecular Genetics, University of TorontoToronto, ON, Canada; 7 Department of Obstetrics & Gynaecology, University of TorontoToronto, ON, Canada

**Keywords:** angiogenesis, diabetic retinopathy, retinopathy of prematurity, Sticky-trap, VEGF

## Abstract

Current therapeutic antiangiogenic biologics used for the treatment of pathological ocular angiogenesis could have serious side effects due to their interference with normal blood vessel physiology. Here, we report the generation of novel antivascular endothelial growth factor-A (VEGF) biologics, termed VEGF “Sticky-traps,” with unique properties that allow for local inhibition of angiogenesis without detectable systemic side effects. Using genetic and pharmacological approaches, we demonstrated that Sticky-traps could locally inhibit angiogenesis to at least the same extent as the original VEGF-trap that also gains whole-body access. Sticky-traps did not cause systemic effects, as shown by uncompromised wound healing and normal tracheal vessel density. Moreover, if injected intravitreally, recombinant Sticky-trap remained localized to various regions of the eye, such as the inner-limiting membrane and ciliary body, for prolonged time periods, without gaining access either to the photoreceptors/choriocapillaris area or the circulation. These unique pharmacological characteristics of Sticky-trap could allow for safe treatment of pathological angiogenesis in patients with diabetic retinopathy and retinopathy of pre-maturity.

## Introduction

Abnormal vessel formation is implicated in the pathophysiology of a number of diseases including diabetic retinopathy (DR), retinopathy of pre-maturity (ROP), the wet form of age-related macular degeneration (AMD), cancer and obesity (Carmeliet, 2003, 2005; Folkman, 2007). Inhibition of pathological angiogenesis has been shown to improve vision in patients with AMD, DR and ROP (Ferrara & Kerbel, 2005; Gasparini *et al*, 2005; Kerbel, 2008; Mintz-Hittner *et al*, 2011), as well as help maintain metabolic homeostasis in obese individuals (Sun *et al*, 2012). Over the past decade, different approaches to inhibit VEGF signalling have been developed, such as recombinant trap proteins (VEGF-trap; Holash *et al*, 2002), monoclonal antibodies (bevacizumab; Ellis, 2005; Ferrara *et al*, 2004) and small multi-kinase inhibitors (Wilhelm *et al*, 2006; Chow & Eckhardt, 2007), some of which have been approved for clinical use (Grothey & Galanis, 2009; Heath & Bicknell, 2009) in treating eye diseases as well as certain types of cancer.

However, the aforementioned approaches may be accompanied by a number of severe side effects due to the disruption of proper vessel function in various organs (Eremina *et al*, 2003, 2006; Kamba *et al*, 2006; Verheul & Pinedo, 2007; Chen & Cleck, 2009; Boehm *et al*, 2010). As a consequence, the use of anti-VEGF therapies may be relatively contraindicated, particularly in certain patient populations, such as pregnant women (Petrou *et al*, 2010), patients undergoing surgery (Cortes *et al*, 2012), pre-mature infants (Hard & Hellstrom, 2011) and elderly patients with comorbidities, such as cardiovascular insufficiency (Chen & Cleck, 2009; Nazer *et al*, 2011).

The storage of numerous growth factors in the extracellular matrix (ECM), as well as the creation of morphogen gradients during development, is controlled by the interaction between the heparin-binding domain (HBD; a sequence of positively charged amino acids) of the growth factor and extracellular-matrix heparan sulphate proteoglycans (HSPGs). VEGF contains two regions with HBDs coded by exons 6 and 7. Alternative splicing of these exons results in distinct isoforms (Robinson & Stringer, 2001; Ladomery *et al*, 2007). VEGF121 is missing both exons and is therefore completely soluble. VEGF145 and VEGF165 contain exon 6 and 7, respectively, and are therefore partially soluble. VEGF189 has both exons and is strictly retained at the secretion site (Houck *et al*, 1992; Park *et al*, 1993).

In order to circumvent the aforementioned disadvantages of current antiangiogenic therapies, we have developed novel biologics with properties that allow for local inhibition of angiogenesis. By genetically fusing the original VEGF-trap (Holash *et al*, 2002) to the HBDs of VEGF, we created novel VEGF-traps, referred to as Sticky-traps, engineered to possess: (i) a short systemic half-life in it's soluble form and (ii) local antiangiogenic activity via the ability to bind to the ECM thus leading to locally retained, biologically active molecules that provide prolonged angiostatic activity at the site of pathological neovascularization.

## Results

### Design and production of VEGF Sticky-traps

**Figure 1:**
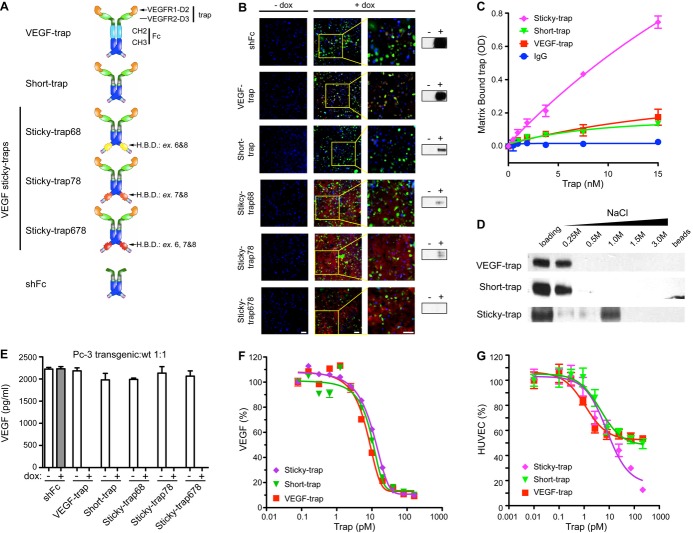
Schematic representation and biochemical characterization of traps. A Basic protein structure of traps. VEGF-trap is composed of the VEGF-binding region (domain 2 and 3 of VEGFR-1 and -2, respectively) and the Fc region of IgG1 (CH2 and CH3 domains). The Sticky-traps, Sticky-trap68, Sticky-trap78 and Sticky-trap678 contain the heparin-binding domains (HBDs) encoded by exons 6 and 8, 7 and 8, and 6, 7 and 8 of vascular endothelial growth factor, respectively. See Supplementary Fig S1A and methods for further details. B–D Affinity of traps to extracellular matrix (ECM). (B) Immunostaining (red signal) and Western blot analysis (on the right of the image) of traps in PC-3 cell monolayers and conditioned supernatant, respectively. Similar results are shown in Supplementary Fig S4A for the A-673 transgenic lines. Plus (+) dox samples were collected 48 h after addition of doxycycline-containing media. Scale bar, 100 μm. (C) Binding of recombinant traps to ECM. (D) Affinity of recombinant traps to heparin-Sepharose column. E, F Assessment of traps ability to bind human VEGF. (E) Free VEGF levels in the conditioned of PC-3 cancer transgenic cell lines, co-cultured of wild-type PC-3 cells. Media were collected after 48 h of culture with or without the addition of doxycycline. (F) Binding affinity (KD) of recombinant traps to human VEGF_165_ (VEGF-trap; 9.0 pM, Short-trap; 12.2 pM, and Sticky-trap 13.9 pM). G Inhibition of VEGF-induced human umbilical vein endothelial cells proliferation by recombinant traps. Source data are available online for this figure.

The original VEGF-trap (Holash *et al*, 2002) is composed of IgG-like domains 2 and 3 of VEGF-R1 and VEGF-R2, respectively, fused to the constant region (Fc) of immunoglobulin IgG1 (Fig 1A and Supplementary Fig S1A). The Fc region is composed of two IgG-like domains, CH2 and CH3, and is responsible for the long serum half-life of VEGF-trap, due to its ability to bind to the FcRn receptor located on endothelial, epithelial and circulating blood cells, after which it is recycled back into the circulation instead of undergoing degradation (Ghetie & Ward, 1997). In order to shorten the half-life, we replaced the CH2 domain, which has been previously shown to be necessary for binding to FcRn (Mueller *et al*, 1990), with a hinge domain (H'; Glaser *et al*, 2005) and a poly-glycine-serine linker (Glaser *et al*, 2005; Fig 1A and Supplementary Fig S1A), and named this molecule Short-trap. To derive Sticky-traps, we modified the Short-trap by adding the HBDs of the VEGF isoforms (HBDs; Munoz & Linhardt, 2004), encoded by exons 6 and 7, at the carboxy-terminus. We also included VEGF exon 8, which is necessary for proper disulphide-bond formation of the HBD (Krilleke *et al*, 2007). In total, we generated three Sticky-traps: Sticky-trap68, Sticky-trap78 and Sticky-trap678, where the numbers indicate which VEGF exons were included (Fig 1A and Supplementary Fig S1A and B). Finally, we generated a “trapless” construct coding only for the short Fc (shFc), as a negative control (Fig 1A and Supplementary Fig S1A).

We then used genetic and recombinant protein-based approaches to characterize these new biologics. In both approaches, we combined the *piggyBac* transposon (Ding *et al*, 2005) transgene delivery system with a tetracycline-based ON/OFF switch (Agha-Mohammadi *et al*, 2004; Supplementary Fig S2A) for transgene expression in a doxycycline (dox)-dependent manner. To test the biologic activity/property of the engineered novel biologics with genetic approaches, we derived transgenic cancer cell lines (PC-3, A-673 and HT-29; Supplementary Fig S2B–D). In the absence of doxycycline (dox), none of the transgenes were expressed, while in the presence of dox, all were expressed at a high level and in comparable amounts (Supplementary Fig S2B–D). We also attached green fluorescent protein (EGFP) as a reporter for transgene expression. Flow cytometry for EGFP in the induced cells showed that on average 88% of the cells expressed the transgene (Supplementary Fig S2E and Supplementary Table S1).

To produce and purify recombinant proteins, we generated stably transfected 293 suspension cell lines using vectors allowing for expression of recombinant traps in a dox inducible manner (Li *et al*, 2013). Of the Sticky-traps, Sticky-trap678 was selected for further characterization, given its superior potency in a xenograft assay (discussed below). All of the traps formed homodimers, had the predicted molecular weight (Supplementary Fig S3A) and were glycosylated (Supplementary Fig S3B).

### Molecular and biochemical characterization of Sticky-traps

Immunostaining, performed on transgenic PC-3 (Fig 1B) and A-673 cells (Supplementary Fig S4A), for the Fc domain showed—as expected—that shFc, VEGF-trap and Short-trap did not bind to the ECM. However, Sticky-traps were bound and retained by the ECM components, indicating that the VEGF HBDs remained functional in these molecules. Further support for the strong affinity of Sticky-traps to the ECM was obtained from Western blot analysis, in which high levels of soluble (free) shFc, VEGF-trap and Short-trap were detected in the culture media, while Sticky-traps were hardly detectable (Fig 1B and Supplementary Fig S4A). Using recombinant proteins, we performed ECM-based and heparin column assays (Fig 1C and D). Sticky-trap showed a dose-dependent ability to bind to the ECM (Matrigel matrix), while VEGF-trap, Short-trap and IgG showed no affinity (Fig 1C). Furthermore, high NaCl concentration was required for elution of Sticky-trap, further indicating its strong affinity for the ECM (Fig 1D).

Sticky-traps also retained their ability to bind VEGF, as confirmed by a competitive ELISA that detects only free VEGF (Maynard *et al*, 2003; Tissot van Patot *et al*, 2005). After addition of dox to the media of transgenic cell lines, both Short-trap and Sticky-traps, similar to VEGF-trap, blocked the appearance of free VEGF in the conditioned media (Supplementary Fig S4B–D). Binding was also shown to be extracellular as VEGF was still completely sequestered from the supernatant when a 1:1 mix of wild-type and transgenic PC-3 cells was assayed (Fig 1E).

We used equilibrium binding assays to determine the binding affinity of recombinant traps for VEGF. Various amounts of traps were incubated with VEGF165, and the free, unbound VEGF165 was measured using a sensitive immunoassay. All traps showed strong affinity for VEGF with similar KD (VEGF-trap; 9.0 pM, Short-trap; 12.2 pM, and Sticky-trap 13.9 pM; Fig 1F). Finally, as a functional assay, we used human umbilical vein endothelial cell (HUVEC) to examine the ability of recombinant traps to inhibit VEGF-induced proliferation and inhibit VEGFR-2 Y1175 phosphorylation. Various amounts of traps were pre-incubated with fixed amounts of VEGF and added to HUVEC cell cultures. All traps were able to efficiently block VEGF-induced proliferation in a dose-dependent manner (Fig 1G; percentage inhibition compared to vehicle only), as well as inhibit VEGFR2 phosphorylation at Y1175 (Supplementary Fig S3C). The enhanced ability of Sticky-trap to inhibit HUVEC proliferation in doses higher than 10 pM is presumably attributed to the fact that both Sticky-trap and VEGF have the same HBD. Therefore, it is possible that Sticky-trap can affect the bioavailability of VEGF by competing for available binding sites in the ECM, as well as compromise the ability of VEGF to bind to neuropilin-1, which is mediated through exons 7 and 8 of the HBD, and is required for VEGFR-2 activation (Parker *et al*, 2012).

### Pharmacokinetics and systemic distribution

**Figure 2:**
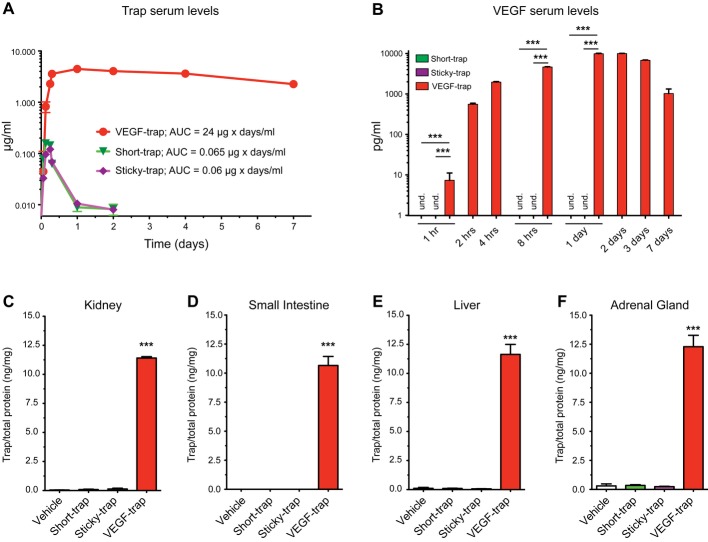
Pharmacokinetic profile and tissue distribution of traps. A Traps (100 μg) were injected subcutaneously into C57BL/6J mice, and serum levels were estimated using an ELISA assay. Error bars represent s.e.m. (*n *=* *5). B Serum levels of VEGF at various time points after subcutaneous injection of traps (100 μg) into C57BL/6J mice. Error bars represent s.e.m. (*n *=* *5; ***P <* *0.001, one-way ANOVA). C–F Trap levels in various tissues 48 h post-subcutaneous injection of traps (100 μg) into C57BL/6J mice. Error bars represent s.e.m. (*n *=* *5; ***P <* *0.001, one-way ANOVA).

In order to characterize the *in vivo* pharmacokinetics of Sticky-trap, after subcutaneous injection, we used an ELISA assay to measure their concentration in the serum. As expected, high amount of VEGF-trap was detected (*C*_max_ = 10 μg/ml; AUC = 24 μg × days/ml), while both Short-trap and Sticky-trap had an AUC that was around 400-fold lower (*C*_max_ = 0.10 μg/ml; AUC = 0.065 μg × days/ml and *C*_max_ = 0.10 μg/ml; AUC = 0.06 μg × days/ml, respectively; Fig 2A), and were practically undetectable 24–48 h after injection.

Previous studies have reported a spike of serum VEGF after treatment with antiangiogenic agents, such DC101 (i.e. anti-VEGF-R2 Ab) and sunitinib (Bocci *et al*, 2004; Ebos *et al*, 2007). Therefore, we examined whether this was also the case for traps. We observed an increase of circulating VEGF in the serum within 1 h post-injection of VEGF-trap, which plateaued between 24 and 48 h (Fig 2B). In the case of Short-trap and Sticky-trap, we did not observe any increase of serum VEGF 1, 8 h and 1 day post-injection, indicating the absence of any systemic activity (VEGF levels were not assayed for the rest of the time points, while VEGF baseline levels in the control (vehicle only) mice were undetectable).

Finally, to exclude the possibility that Sticky-trap due to its high affinity to the ECM is deposited in tissues, we examined the distribution in various tissues and especially those with fenestrated vessels. Two days post-subcutaneous injection, we showed that in contrast to VEGF-trap, Short-trap and Sticky-trap were undetectable in kidney, small-intestine, liver and adrenal gland (Fig 2C–F).

### Sticky-traps inhibit angiogenesis *in vivo* and remain at the site of introduction

**Figure 3:**
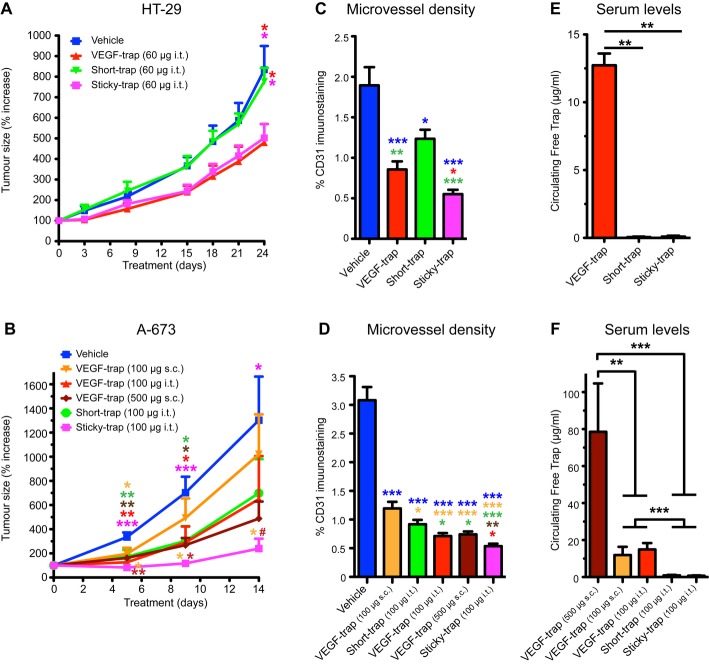
Characterization of recombinant traps in vivo antiangiogenic activity in xenograft models. A Tumour growth kinetics of HT-29 xenografts. Treatment (30 μg per tumour, intratumorally injected; two-times per week) initiated 1 week after tumour implantation, once tumours reached an average size 100 mm^3^. Error bars represent s.e.m. (*n *=* *12; *P <* *0.05, one-way ANOVA). B Tumour growth kinetics of A-673 xenografts. Treatment (50 μg per tumour, intratumorally injected, or 100/500 μg subcutaneously injected; three times per week) initiated 1 week after tumour implantation, once tumours reached an average size of 50 mm^3^. Error bars represent s.e.m. (*n *=* *8; ***P < 0.001, **P <* *0.01, *P <* *0.05, one-way ANOVA). C, D Microvascular density of HT-29 and A-673 xenografts, respectively. Paraffin sections (Supplementary Figs S8 and S9) were immunostained with an anti-CD31 antibody (***P <* *0.001, **P <* *0.01, *P <* *0.05, one-way ANOVA). E, F Circulating levels of free traps. Serum from HT-29 (E) and A-673 (F) tumour-bearing mice, treated with various traps, was collected at day 24 and 14, respectively (***P <* *0.001, **P <* *0.01, one-way ANOVA). Source data are available online for this figure.

Although our intention here is to propose the use of Sticky-traps as therapeutic agents to suppress pathological neovascularization in eye diseases, in order to initially explore and evaluate the *in vivo* effect of Sticky-traps with ease, we used tumour xenograft assays (Fig 3 and Supplementary Figs S5–S12). Nude mice were used as recipients for subcutaneous xenografts, and transgenic expression of traps (and shFC control) was induced with dox-containing food (*dox*-chow). Expression was visualized by EGFP. Prior to dox administration, none of the xenografts expressed EGFP, while expression was detected as early as 2 and 4 days post-administration and reached a plateau at day 4 and 8, in the A-673 and HT-29 xenografts, respectively (Supplementary Figs S6A and S7A). EGFP expression by PC-3 cancer lines was very weak (data not shown).

Upon trap expression, the PC-3 derived xenografts significantly decreased in size, some completely regressed (Supplementary Fig S5A) with only small foci remaining (Supplementary Fig S5B). Tumour-stasis was observed in HT-29 xenografts expressing Sticky-trap678, while a small increase in tumour size was observed when VEGF-trap was induced. Likely due to its short half-life, Short-trap was the least effective; tumours expressing this variant kept growing, albeit at a much slower rate compared to the shFc control (Supplementary Fig S6B). A-673-derived xenografts stopped growing and the average tumour volume remained constant during the treatment (Supplementary Fig S7B). The stasis of the tumour was independent of the initial volume of the A-673 xenograft tumours (data not shown).

The results from the HT-29 xenografts indicated that Sticky-trap678 was the most efficient trap; thus, we produced recombinant protein corresponding to this isoform. To characterize the ability of the recombinant traps to inhibit tumour progression, we established xenografts using wild-type HT-29 and A-673 cells (Fig 3A and B). HT-29-derived tumours were not affected by intratumourally injected Short-trap (Fig 3A), while Sticky-trap resulted in tumour growth delay, similar to VEGF-trap (Fig 3A).

In our A-673 xenograft studies, we also performed systemic delivery of recombinant VEGF-trap via subcutaneous injections. Sticky-trap was able to almost completely inhibit tumour progression and was significantly more efficient than equal amounts of systemically administered VEGF-trap (VEGF-trap 100 μg s.c., *P *<* *0.01; Fig 3B). Intratumourally injected Sticky-trap was also initially more efficient than a 5-times higher dose of systemically administered VEGF-trap, losing statistical significance between day 9 and 14 (*P *=* *0.0048 at day 5, *P *=* *0.0343 at day 9, and *P *=* *0.1857 at day 14).

We then analysed the effect of trap expression on tumour microvascular density by immunostaining of xenografts with antibodies recognizing the endothelial marker CD31 (PECAM). Although all traps dramatically decreased tumour microvascular density (Fig C and D, and Supplementary Figs S6C, S6D, S7C, S7D, S8 and S9), recombinant Sticky-trap was the most potent (Fig C and D). The vessel-density decrease resulted in hypoxic regions in the HT-29 tumours (Supplementary Fig S6D), and extensive areas of necrosis in both A-673 and HT-29 tumours (Supplementary Figs S6E and S7E).

To examine the levels of the traps in the systemic circulation, we collected serum from tumour-bearing mice treated with recombinant traps and measured the levels of free circulating traps using a functional ELISA. Circulating VEGF-trap could be detected in both xenograft models— HT-29 and A-673—while Short-trap and Sticky-trap were undetectable (Fig E and F). We observed a similar pattern in the case of xenografts using transgenic cancer cell lines (Supplementary Figs S10 and S11). We also collected urine sample from mice bearing xenografts expressing either VEGF-trap, Short-trap or Sticky-traps. By Western blotting, we detected high amount of intact VEGF-trap in urine of mice with either A-673 or HT-29 xenograft (Supplementary Fig S12A and B), while only fragments of Sticky-trap678 were detected in urine of mice with A-673 xenografts (Supplementary Fig S12A).

### Sticky-traps have a favourable toxicity profile

**Figure 4:**
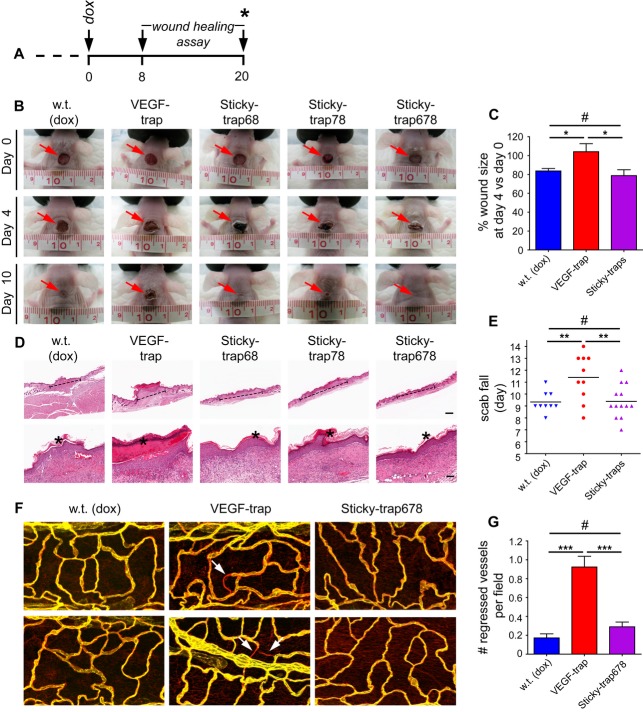
Wound-healing assay and detection of regressed vessels in the trachea. A Wounds were generated at day 8 in the neck area of mice bearing xenografts of transgenic A-673 expressing either VEGF-trap or Sticky-traps. Wound size and spontaneous fall of the scab were monitored daily. B–E At day 20, a different group of animals was euthanized and the trachea was dissected (A, asterisk). Serum was collected at day 0, 8 and 20 (A, arrows; Supplementary Fig S11). (B and C) Wound size at day 4 compared to day 0 (*P <* *0.05, ^#^ *P *>* *0.05, one-way ANOVA). (B, D and E) Spontaneous scab fall (**P <* *0.01, ^#^ *P *>* *0.05, one-way ANOVA; *n *=* *9 for wild-type, *n *=* *10 for VEGF-trap, and *n *=* *13 for Sticky-traps; *n *=* *4 for Sticky-trap68, *n *=* *4 for Sticky-trap78, *n *=* *5 for Sticky-trap678). (D) H&E staining of representative wounds. Dashed line indicates the wound area, and asterisk the scab. Scale bars, 500 μm (top row) and 50 μm (bottom row). F, G Immunostaining of tracheal vessels for CD-31 (yellow) and type-IV collagen (red). Arrows indicate regressed (ghost) vessels, that is, vessels of type-IV collagen immunoreactivity devoid of CD31 immunoreactivity (***P =* *0.0008 for w.t. versus VEGF-trap and *P *=* *0.0009 for Sticky-trap678 versus VEGF-trap, ^#^ *P *>* *0.05, one-way ANOVA; *n *=* *4 for w.t., *n *=* *5 for VEGF-trap and *n *=* *5 for Sticky-trap678).

As mentioned previously, systemic anti-VEGF therapies may be associated with adverse side effects. We therefore investigated the potential systemic effects of Sticky-traps outside the tumour environment of A-673 xenograft-bearing animals. At a tumour size of 1.5 cm^3^, transgene expression was induced, and 8 days later, full-thickness excisional wounds were created in the neck area of the animals (Niethammer *et al*, 2002; Seavey *et al*, 2009; Fig 4A). Consistent with the known wound healing delay following systemic VEGF suppression (Bose *et al*, 2010), 4 days after wounding, the wound size of animals with tumours expressing the soluble VEGF-trap was significantly larger than those expressing Sticky-trap (Fig 4B and C; *P *<* *0.05). Animals with tumours expressing Sticky-traps and control non-tumour-bearing animals fed with *dox*-chow showed the same wound size (Fig 4B and C; *P *>* *0.05). Similarly, a longer time period was required for the scab to fall off from the wounds on animals bearing tumours expressing VEGF-trap compared to control animals and those expressing Sticky-traps (Fig 4B, D and E; *P *<* *0.01). No difference was observed between the latter two (Fig 4B, D and E; *P *>* *0.05).

In a second model, we also measured systemic VEGF suppression by examining tracheal micro-vessels (Kamba *et al*, 2006) by whole-mount immunostaining with antibodies recognizing endothelial cells (anti-CD31) and their basement membrane (anticollagen IV). Functioning vessels are double positive, while vessel regression leads to areas positive for collagen IV and negative for CD31 (called empty sleeves; Kamba *et al*, 2006). Animals with tumours expressing Sticky-trap678 and non-tumour-bearing controls had very few empty sleeves and the majority of the vessels were double positive (Fig 4F and G; *P *>* *0.05). In contrast, animals with tumours expressing the VEGF-trap showed a fivefold increase in empty sleeve formation (Fig 4F and G; *P *<* *0.001).

**Figure 5:**
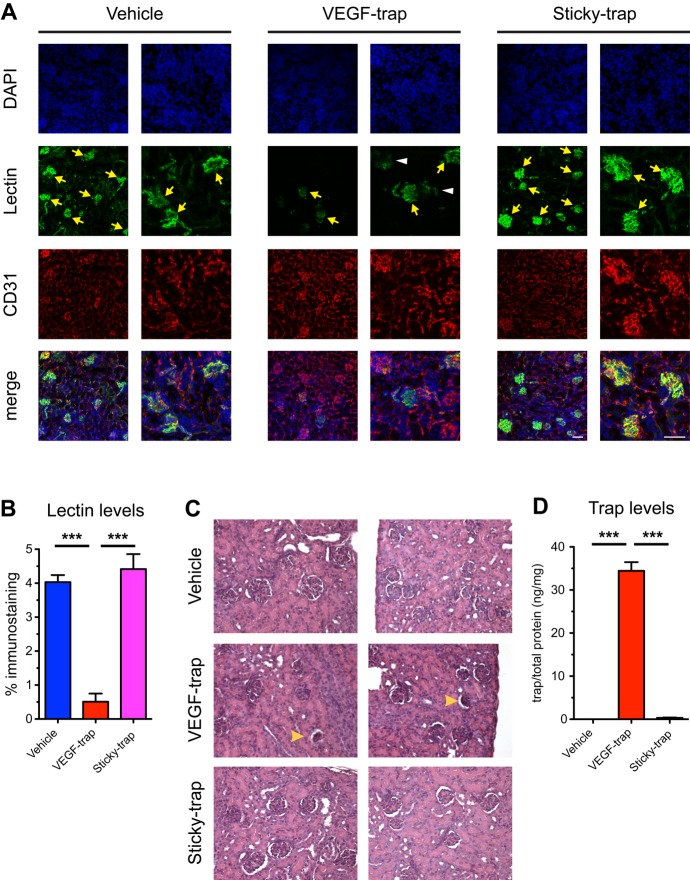
Subcutaneous injection of traps in neonatal pups. Immunostaining of kidneys for vessels (CD31) and perfusion (lectin). Yellow arrows; perfused glomeruli, white arrowheads; partially perfused glomeruli. Scale bars, 50 μm. Quantification of lectin perfusion (***P <* *0.001, one-way ANOVA; *n *=* *5 for vehicle, *n *=* *8 for VEGF-trap and *n *=* *7 for Sticky-trap). Paraffin sections of kidneys. Yellow arrowheads, collapsed glomeruli. Trap levels in kidneys (***P <* *0.001, one-way ANOVA; *n *=* *4 for vehicle, *n *=* *5 for VEGF-trap and *n *=* *5 for Sticky-trap).

In addition, we also used recombinant proteins in order to further examine the safety of Sticky-trap. Due to the limited amount of recombinant Sticky-trap, we decided to use pups and examine the functionality of kidney glomeruli vessels after treatment via lectin immunostaining (Fig 5). In order to model systemic administration, traps (25 mg/kg) were given subcutaneously once every 3 days, starting at P3, and the mice were euthanized after three injections at P12. We observed decreased perfusion of glomeruli vessels in pups treated with VEGF-trap (Fig 5A and B), which caused some of the glomeruli to collapse (Fig 5C), while the glomeruli of pups treated with Sticky-trap appeared normal. We also detected high levels of VEGF-trap in kidney tissue lysates, while Sticky-trap was undetectable (Fig 5D).

Taken together, these results suggest that (i) systemic VEGF antagonism has adverse effects on the maintenance of normal vessels and (ii) no such adverse events are observed with the Sticky-trap.

### Sticky-trap binds to various anatomical areas of the eye

**Figure 6:**
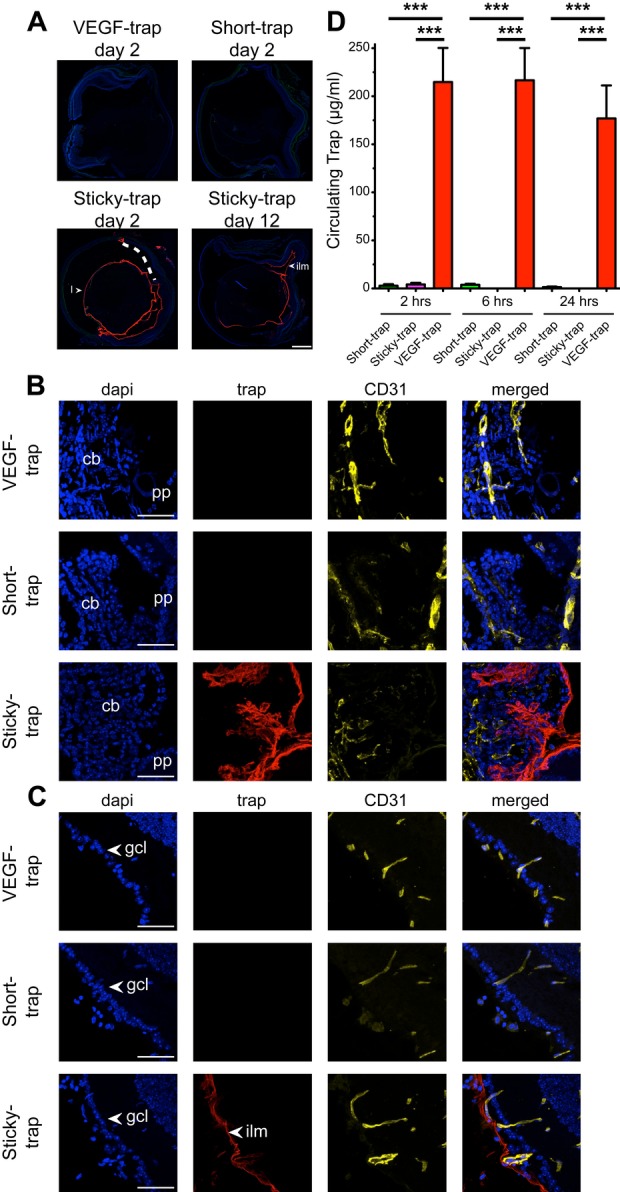
Biodistribution of traps in the eye. A Immunostaining of cross sections of mouse eyes, intravitreally injected with the traps (10 μg) and dissected either 2 or 12 days post-injection. Scale bar, 500 μm. B, C Binding of traps in the ciliary body (B), and inner-limiting membrane (C) of the eye. Scale bar, 50 μm. More detailed immunostaining analysis is shown in Supplementary Fig S13. L, lens; ilm, inner-limiting membrane; cb, ciliary body; gcl, ganglion cell layer. D Traps' serum levels 2, 6 and 24 h post-intravitreal injections. (*n *=* *4; **P <* *0.01, *P <* *0.05, one-way ANOVA).

Given the clinical value of antiangiogenic molecules in the treatment eye diseases and the demonstrated strong retention of Sticky-trap at the site of introduction, we characterized its distribution in the eye after intravitreal injection using immunostaining of cross-sectioned whole eyes. Two days post-injection, only Sticky-trap could be detected in all eyes, while VEGF-trap and Short-trap were not present by then (Fig 6A). Sticky-trap remained in the eye at least for 12-days post-injection (Fig 6A). In contrast to VEGF-trap and Short-trap, Sticky-trap specifically localized to the lens, the inner-limiting membrane, the ciliary body and the ciliary body zonula fibres (Fig 6B and C and Supplementary Fig S13–S16). This distribution was in part overlapping with collagen IV (Supplementary Fig S13). Small amounts of Short-trap and VEGF-trap could be detected in the choroid of some eyes analysed 2 and 8 h post-injection (Supplementary Figs S14A and S15A). Since Sticky-trap was not detected posterior to the inner-limiting membrane, we also performed subretinal injections. Three days post-injection, we could detect Sticky-trap in the subretinal space, between the retinal pigment epithelium (RPE) and outer nuclear layers (Supplementary Fig S17). We also examined the circulating levels of traps after intravitreal injections. Short-trap and Sticky-trap were undetectable, while VEGF-trap was present at a significant level (Fig 6D).

### Sticky-trap efficiently inhibits eye neovascularization

**Figure 7:**
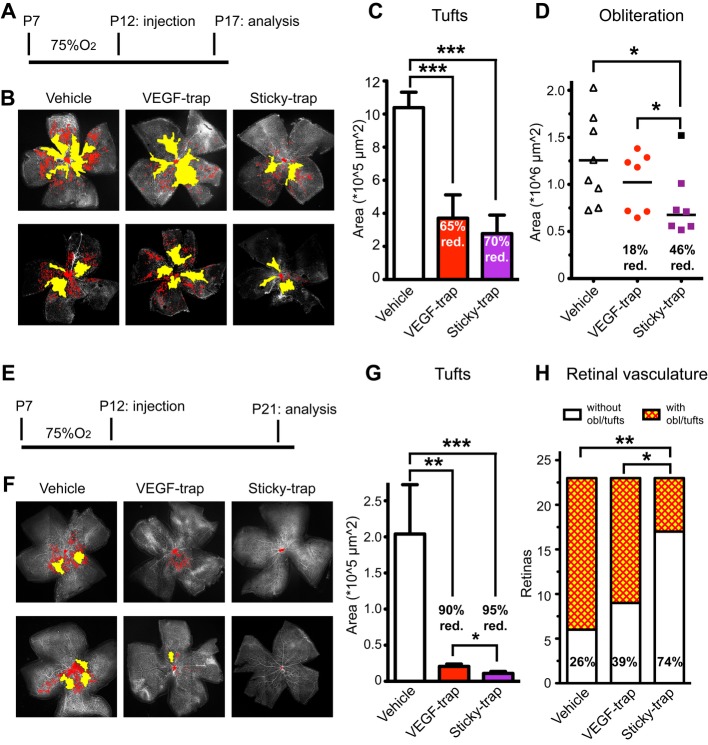
*In vivo* characterization of trap activity in the mouse model of oxygen-induced retinopathy (OIR). A–H Pups were exposed to hyperoxia for 5 days, P7-P12, and traps (2.5 μg) were injected intravitreally at P12, once the mice were returned to normoxia. Eyes were dissected either 5 or 9 days post-injection, at P17 (A–D) and P21 (E–H), respectively. (B and F) Whole-mount immunostaining of retinas for neovascular tuft formation (lectin-positive signal, red pseudocolour) and persisting vaso-obliteration (yellow pseudocolour). (C and G) Area of tuft formation at P17 and P21, respectively (*n *=* *7–8; ***P <* *0.001, *P <* *0.05, one-way ANOVA). (D) Area of retinal obliteration at P17 (*n *=* *7–8; *P <* *0.05, one-way ANOVA; the value coloured in black from the Sticky-trap group was excluded from the analysis). (H) Quantification of retinas with completely normal vascular network, that is absence of obliteration and/or tufts at P21 (*n *=* *23; **P <* *0.01, *P <* *0.05, chi-square test).

The ability of Sticky-trap to specifically bind to the inner-limiting membrane prompted us to study its efficacy in an oxygen-induced retinopathy (OIR) mouse model for DR and ROP. We examined the ability of traps to inhibit ocular neovascularization after intravitreal injections in the eyes of mice exposed to high oxygen from P7 until P12 followed by return to normoxia; one eye was injected with trap, while the contralateral eye served as vehicle control. Pathological vascularization was quantified by immunostaining at either P17 (Fig 7A–D) or P21 (Fig 7E–H). At P17, we found a 65 and 70%, reduction of tuft formation by VEGF-trap and Sticky-trap, respectively (Fig 7B and C). Persisting vaso-obliteration (i.e. avascular area) was reduced by 46% by Sticky-trap compared to 18% when VEGF-trap was used (Fig 7B and D). At P21, Sticky-trap reduced tuft formation by 95%, and VEGF-trap by 90% (Fig 7F and G). Moreover, 74% of the eyes treated with Sticky-trap had no obliteration and/or tufts at all, compared to 39 and 26% in the case of VEGF-trap and vehicle, respectively (Fig 7F and H). Immunostaining for traps showed that Sticky-trap remains in the eye after injection and localizes to the inner-limiting membrane and the ciliary body (Supplementary Fig S18A). We observed the same results when traps were injected at P7 (pre-high oxygen exposure) and analysed at P17 (Supplementary Fig S18B). We also examined the level of circulating traps, and in contrast to VEGF-trap, Sticky-trap was undetectable in the serum (Supplementary Fig S18C).

**Figure 8:**
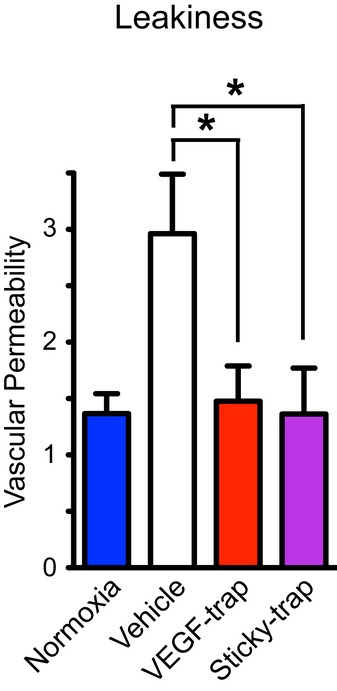
Vascular leakage quantified by Evans Blue dye accumulation in the retina of mice exposed to hyperoxia. *n *=* *8 for vehicle, VEGF-trap and Sticky-trap; *n *=* *3 for normoxia; *P < 0.05, one-way ANOVA.

Finally, we assessed vascular leakage using intravenous Evans Blue injection. Both Sticky-trap and VEGF-trap were able to inhibit vascular leakiness and reduce the levels of Evans Blue in the vitreous to the same level found in wild-type eyes under normoxia (Fig 8).

### Sticky-trap is not toxic to the eye

**Figure 9:**
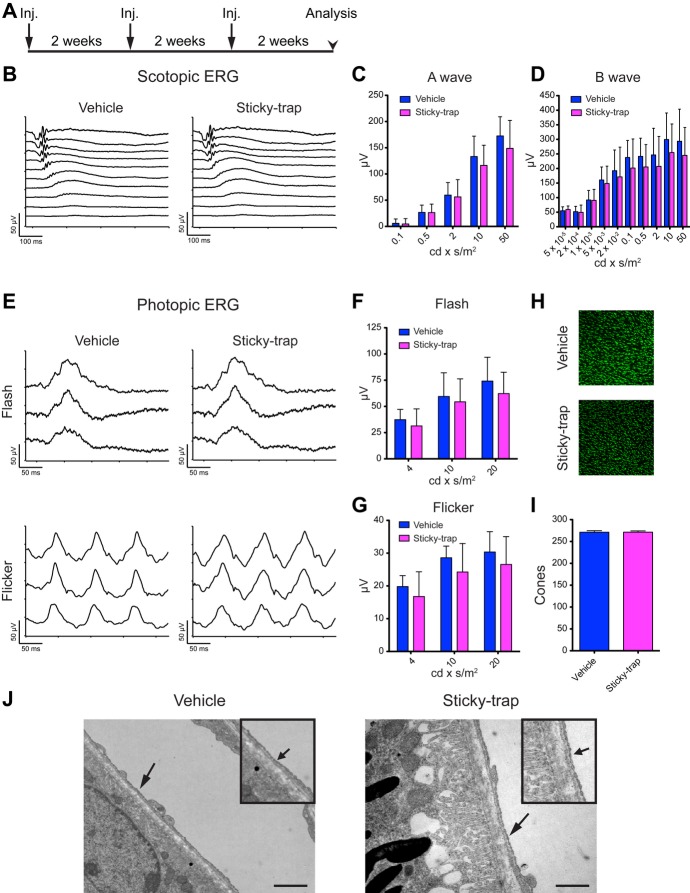
Effect of Sticky-trap in vision after intravitreal injection. A Sticky-trap (2.5 μg) were intravitreally injected three times, with 2-week intervals, into the right eye of C57BL/6J. The contralateral eye was injected with vehicle. B–G The function of rod and cone photoreceptors was evaluated with scotopic (B–D) and photopic (E–G) electroretinographys, respectively, after 6-weeks of treatment. No reduction of either A- or B-wave was observed indicating the normal function of rod photoreceptors (C, D) (*n* = 8). Similarly, both b-wave amplitudes from flash and first peaks from flicker appeared normal (F, G), indicating the normal function of cone photoreceptors (*n* = 8). H, I Quantification of cone outer segments by immunohistochemistry for opsin; there was no difference between Sticky-trap and vehicle (*n* = 5). J Electron micrograph of a cross-sectioned retina from mice treated with either Sticky-trap or vehicle. The choroidal vessels appeared normal (arrows). Scale bars, 1 μm.

Using genetic models, it has previously been reported that deletion of VEGF in adult RPE cells rapidly leads to the ablation of choriocapillaris, pronounced loss of the outer segments of cone photoreceptors and as a consequence to blindness (Nishijima *et al*, 2007; Kurihara *et al*, 2012). In this study, we performed three intravitreal injections of Sticky-trap, over a 6-week span and then examined the function of photoreceptors using electroretinography (ERG; Fig 9A). Using both scotopic and photopic ERGs, we demonstrated that the function of rod (a-wave; scotopic ERG and flicker; photopic ERG) and cone (flash; photopic ERG) photoreceptors was not affected (Fig 9B–G). In addition, the unaltered b-wave amplitude of scotopic ERG indicates that the function of the cells of the inner nuclear layer, such as bipolar and Muller cells, as well as the function of ganglion cells was also not affected (Fig 9D; Dong & Hare, 2000; Bui & Fortune, 2004). Similar results were also observed 1 week after a single subretinal injection of Sticky-trap (Supplementary Fig S19).

Furthermore, using immunohistochemistry to stain for cone photopigment opsin, we showed that there was not any difference in the number of cone photoreceptor outer segments between vehicle and Sticky-trap treated mice (Fig 9H and I). Finally, using electron micrographs, we examined the integrity of the choriocapillaris and showed that it was not affected (Fig 9J). Therefore, we could conclude that the extended presence of Sticky-trap in the eye does not interfere with retina function.

## Discussion

In this study, we employed both genetic and pharmacological approaches to characterize novel anti-VEGF biologics: VEGF Sticky-traps. In contrast to VEGF-trap (Holash *et al*, 2002) that has a broad systemic activity, Sticky-traps are locally retained at the delivery site for an extended amount of time by binding to the ECM; this feature results in a safer toxicity profile. The ability of Sticky-traps to remain at the site of delivery was achieved by engineering the original VEGF-trap to contain a strong HBD and thus to be able to bind to negatively changed heparan sulphate glycosaminoglycans of various HSPGs of the ECM and cell surface. Furthermore, Sticky-traps are at least as effective as VEGF-trap in inhibiting pathological angiogenesis.

Currently approved angiostatic treatments of neovascular eye diseases require frequent intravitreal injections of the eye using expensive drugs (Stewart, 2012). Longer acting compounds, and the use of sustained delivery devices, would allow for less frequent administration and as a consequence could lower the costs and risks associated with repeated intraocular injections. Recent clinical trials have provided evidence that VEGF-trap administered every other month in patients with AMD can be as effective as monthly ranibizumab (Stewart *et al*, 2012a); likely due to its longer predicted intravitreal half-life (4.8 days versus 3.2 days; Stewart *et al*, 2012b) and its higher VEGF-binding affinity (Papadopoulos *et al*, 2012). In the case of bevacizumab, various approaches have been evaluated with the goal of increasing the intravitreal half-life (Pan *et al*, 2011). In this study, we showed that Sticky-trap was more effective compared to VEGF-trap in inhibiting tuft formation and vaso-obliteration in a mouse model of DR and ROP. Moreover, Sticky-trap remained in the eye up to 12 days, which is longer than the time periods reported in the literature for biologics targeting ocular abnormal angiogenesis. This extended retention strongly indicates that Sticky-trap might allow for administration in less frequent intervals.

We provide compelling evidence that in contrast to VEGF-trap, Sticky-trap binds to various areas of the eye, including the inner-limiting membrane after intravitreal injections. Thus, Sticky-trap would be very appealing for treatment of patients with diseases such as DR and ROP, in which new vessels emerge from the retinal vascular network, penetrate the inner-limiting membrane (ILM) and advance into the posterior hyaloid face (Antonetti *et al*, 2012). Previous studies have shown that both the thickness of the ILM and the amount of HSPGs increase with disease progression in the case of DR (Hanneken *et al*, 1991; Matsunaga *et al*, 2005). The later will allow for even higher retention of Sticky-trap in patients with advanced disease, which may result in higher clinical benefit. We were also able to show that after subretinal injections, Sticky-trap binds and remains in the subretinal space; the origin of new vessels observed in AMD (Jager *et al*, 2008). This retention property could allow local AMD treatment with delivery of Sticky-trap in the RPE layer with various methods, such as slow-release implants (Janoria *et al*, 2007), liposomes (Abrishami *et al*, 2009), viruses (Pechan *et al*, 2009) and RPE cells (Otani *et al*, 2002).

Delivery of soluble VEGF antagonists can be worrisome due to the high risk of severe side effects, especially since many of the elderly patients with DR and AMD might have coexisting risk factors (Stewart, 2012). The local activity and short systemic half-life of Sticky-trap may prove especially helpful in these cases. Moreover, pre-mature infants suffering from ROP could also greatly benefit from this novel biologic. Treatment of ROP typically occurs during the neonatal stage (between 30 and 40 weeks post-menstrual age), during organogenesis. During this period, it has been shown that VEGF is very important for the proper development of both kidneys and lungs; its inhibition can lead to dysfunction of glomerular filtration and bronchopulmonary dysplasia (Eremina *et al*, 2006; Thebaud, 2007). In the recent BEAT-ROP clinical trial comparing intravitreal bevacizumab to conventional diode laser photocoagulation for ROP treatment, increased mortality was observed in the bevacizumab versus laser group (6.6 versus 2.6%). Interestingly, four of five deaths in the bevacizumab group (and one of two in the laser group) were due to pulmonary complications (Mintz-Hittner *et al*, 2011). Recent reviews point out that systemic VEGF suppression in pre-mature infants can be detrimental, and strongly suggest cautious and limited use of bevacizumab for this condition (Hard & Hellstrom, 2011; Avery, 2012; Darlow *et al*, 2013). Given the similar pharmacokinetic profile of VEGF-trap, and its even higher affinity, it is reasonable to assume that similar concerns will arise. In contrast, the Sticky-trap could provide a safer therapy for ROP since following intravitreal injections; it does not escape into the circulation.

In this study, we characterized our novel bifunctional antiangiogenic biologic, the Sticky-trap, and demonstrated its efficacy in neovascularization models. Due to its unique property to locally inhibit abnormal angiogenesis, Sticky-trap merits further exploration as a key component of antiangiogenic therapies.

## Materials and Methods

### Construction of VEGF-traps and *piggyBac* expression system

Traps were generated using basic molecular biology techniques. VEGF-trap (1479 bp; 492 a.a.; M.W. 54.8 kDa) is composed by (i) the signal peptide (NP_002010, a.a. 1–31), (ii) domain-2 of human VEGFR-1 (NP_002010, a.a. 131–231), (iii) domain-3 of human VEGFR-2 (NP_002244, a.a. 226–327) and (iv) the Fc region of human IgG1 (H domain; P01857.1, a.a. 99–113, plus CH2 domain; P01857.1, a.a. 114–223, plus CH3 domain; P01857.1, a.a. 224–330). Two epitope tags (FLAG: DYKDDDDK and His: HHHHHHHH) were added to the carboxy-terminus with GS1 linkers (GGGS) in between. For the generation of Short-trap (1227 bp, 408 a.a., M.W. 44.8 kDa), the CH2 domain was substituted by (i) a H' domain (17 a.a.; EPKSCDTPPPCPRCPAR; Glaser *et al*, 2005) and (ii) a GS2 linker (GGGSSGGGS; Glaser *et al*, 2005).

For the generation of the Sticky-traps (Sticky-trap68: 1332 bp, 443 a.a., M.W. 51.0 kDa; Sticky-trap78: 1392 bp, 463 a.a., M.W. 51.0 kDa; and Sticky-trap678: 1464 bp, 487 a.a., M.W. 54.0 kDa), the mouse vascular endothelial growth factor (VEGF-A) exons 6 (NP_001020421, a.a 319–342), 7 (NP_001020421, a.a 343–386) and 8 (NP_001020421, a.a 387–392) were used, and a GS3 linker (GGGAS) was placed between the CH3 and VEGF-A exons.

The transposons, PB-TAG and PB-rtTA, were generated by modifying PB-TET (Woltjen *et al*, 2009) and pcDNA-rtTA/neo (IMP, AN unpublished). PB-TAG is composed of the second-generation tetracycline-regulated promoter (TRE; Agha-Mohammadi *et al*, 2004) driving the expression of the transgene coupled to EGFP through an internal ribosomal entry site (IRES). PB-rtTA is composed of the reverse-transactivator of tetracycline (rtTA; Urlinger *et al*, 2000) and the neomycin resistance gene, both driven by separate constitutive promoters.

### Cell culture and generation of cancer cell lines

The human prostate cancer PC-3, human rhabdomyosarcoma A-673 and human colon adenocarcinoma HT-29 cell lines were purchased from ATCC and maintained in RPMI-1640, DMEM and McCoy's 5A media, respectively, all containing 10% foetal bovine serum.

Briefly, for the generation of stable cell lines, 1 × 10^6^ cells were plated per well (9.6 cm^2^/well) in a 6-well plate and were transfected 16 h after with 0.5 μg of PB-rtTA/neo and 2.5 μg of PB-TAG-GOI along with 0.5 μg PBase using ExGen500 (Fermentas, cat. # R0511) according to the manufacturer's protocol. Stable clones were derived after 2 weeks of selection using 750 μg/ml G418. To simplify the method and maintain a heterogeneous cancer cell population, all G418 resistant cells were trypsinized and pooled together. For induction of gene expression, we used 2 μg/ml of doxycycline (Sigma, cat. # D9891) in the culture media. Cell lines were cultured at 37°C, in 100% humidity and 5% CO_2_ containing atmosphere.

### Generation of mammalian cell lines for protein expression

Stable mammalian cell lines expressing recombinant proteins were established as has been previously described (Li *et al*, 2013).

### Protein purification

The harvested media containing the secreted proteins were concentrated 10- to 20-fold on a Prep/Scale Spiral Wound 10-KDa MWCO Ultrafiltration module (Millipore, cat. # SK1P003W4). Recombinant proteins were purified from the concentrated media by immobilized-metal affinity chromatography on Ni-NTA columns (Bio-Rad, cat. # 156-0135). Purified proteins were quantified using absorbance at A280 nm (NanoDrop UV-Vis spectrophotometer; Thermo Scientific) and confirmed by Coomassie blue staining after SDS–PAGE.

### Protein isolation

For isolation of cytoplasmic and secreted protein from stably transfected cancer lines, 1 × 10^6^ cells were plated per well (9.6 cm^2^/well) in a 6-well plate and cultured with and without doxycycline for 48 h. Supernatants were collected, centrifuged and stored at −70°C. Subsequently, the cytoplasmic proteins along with ECM proteins of the cell monolayer were isolated using RIPA lysis buffer containing protease inhibitors (Roche, cat. # 11836170001). Plates containing 250 μl of lysis buffer per well were incubated for 30 min on ice. The lysis was collected and stored at −70°C.

Protein from xenografts was isolated from frozen tumour sections. Around a half gram of tissue was quickly homogenized on ice in 3 ml ice-cold RIPA lysis buffer containing protease inhibitors mentioned above using homogenizer. Homogenates were incubated for 1 h at 4°C and centrifuged at 15,000 × *g* for 30 min at 4°C. The supernatant was collected and frozen at −20°C. Aliquots of supernatant were collected for protein determination by the Bradford method (Bio-Rad protein assay).

### Western blot assays

Cell culture extracts and supernatants, tumour protein extracts and plasma were resolved by 4–20% SDS–PAGE and transferred to nitrocellulose membranes. Membranes were blocked in 5% non-fat milk in TBS-T buffer (10 mM Tris pH 7.5, 150 mM NaCl and 0.1% Tween 20). A goat anti-human Fc IgG1-HRP-conjugated antibody (1 in 5,000; Jackson Immunoresearch, cat. # 109-035-098) was used for detection of VEGF-traps. Loading for cell culture and tumour extracts was assessed with rabbit antibody against human beta-actin (1 in 10,000; Sigma, cat. # A5441) followed by anti-rabbit IgG1-HRP-conjugated antibody (1 in 10,000; Bio-Rad, cat. # 170-6515).

### VEGFR2 tyrosine phosphorylation assay

Human umbilical vein endothelial cells grown to confluency were starved in serum-free media overnight and then treated with 1–10 μg/ml VEGF-trap inhibitors for 2 h at 37°C prior to the tyrosine phosphorylation assay. Cells were pre-treated with 200 μM Na3VO4 in serum-free media for 5 min at 37°C and then exposed to 100 ng/ml VEGF, which was pre-incubated with and without inhibitors for 30 min prior to use, in serum-free media for 5 min 37°C. Cells were then lysed in modified RIPA (mRIPA) buffer (50 mM Tris, pH 7.4, 150 mM NaCl, 1% NP-40, 0.25% sodium deoxycolate, 1 mM EDTA, 1 mM sodium orthovanadate, 10 mM β-glycerophosphate and protease inhibitors [1 mM phenylmethanesulfonyl fluoride (PMSF), 20 μg/ml leupeptin, and 20 μg/ml aprotinin]) and evaluated by immunoblot analysis using the following antibodies at a dilution of 1:1000: rabbit anti-VEGFR2 (55B11) (Cell Signaling, Danvers, MA, USA), rabbit anit-phospho-VEGFR2 Y1175 (Cell Signaling) and mouse anti-GAPDH (EMD Millipore, Billerica, MA, USA).

### Flow cytometry analysis

For flow cytometry analysis, 1 × 10^6^ cells were plated per well (9.6 cm^2^/well) in a 6-well plate and cultured with or without doxycycline for 48 h, trypsinized and suspended into PBS containing 1% v/v of 7-AAD (BD Pharmingen, cat. # 559925) for detection of apoptotic cells. The FACSAria™ cell sorter (BD Biosciences) was used for single cell analysis.

### ELISA assays

Enzyme-linked immunosorbent assay for VEGF-trap was developed as has been previously described (Koh *et al*, 2010). A 96-well plate was coated overnight at 4°C with 200 ng of VEGF in 100 μl of PBS. The plate was washed four times with 400 μl wash buffer [PBS, 10% SBlock (Pierce, cat. # 37538), 0.05% Tween-20] and then coated for 2 min with SBlock at RT. Fifty microlitres of trap standards, samples and controls were added along with 50 μl of incubation buffer (100% SBlock, 0.1% Tween-20) and incubated on the bench, at RT for 2 h. After washing four times with 400 μl of wash buffer, 50 μl of HRP-conjugated goat anti-human IgG-Fc (1:10,000 dilution in PBS; Jackson Immunoresearch, cat. # 109-035-098) along with 50 μl of incubation buffer was added in each well and the plate was incubated at RT for 2 h. The plate was washed four more times with 400 μl of wash buffer, before addition of 50 μl of 3,3′,5,5′-tetramethylbenzidine (TMB) solution (Thermo Scientific, cat. # 34028) and incubation at RT for 30 min. The reaction was stopped with stopping solution (Sigma, cat. # S5814), and the absorbance at 450 nm was measured using an ELISA reader (Bio-Rad).

Human VEGF was measured using a commercially available sandwich ELISA (R&D systems, cat. # DVE00) following the manufacturer's protocol. One hundred microlitres of tissue supernatant was used for each sample.

### VEGF-binding assay

Binding affinity of traps was determined using an ELISA able to detect unbound human VEGF (R&D Systems, cat. # DVE00). Various concentrations of traps (0.15–160 pM) were incubated with 10 pM VEGF_165_ (R&D Systems, cat. # 293-VE-010) and resuspended at final concentration 0.1 μg/μl in PBS, 1 mg/ml Fraction V BSA) in incubation buffer (50 mM phosphate buffer, 150 mM NaCl, 1 mg/ml fraction V BSA, pH 7.5) overnight at room temperature. Low-binding tubes were used for the incubation. For the ELISA assay, 200 μl of each incubation sample was added per each well containing 50 μl of assay diluent RD1W.

### Matrigel-based ECM binding assays

Matrigel-matrix-coated plates (BD Biosciences, cat. # 354607) were incubated with 100% SBlock (Pierce, cat. # 37538), for 10 min at room temperature. One hundred microlitres of varying amounts recombinant proteins diluted in 50% of Sblock in PBS was added and incubated at RT for 2 h. After washing four times with 400 μl of wash buffer (10% SBlock, 0.1% Tween-20 in PBS), 100 μl of HRP-conjugated goat anti-human IgG-Fc (1:10,000 dilution in 50% of SBlock in PBS; Jackson Immunoresearch, cat. # 109-035-098) was added in each well and the plate was incubated at RT for 2 h. The plate was washed four more times with 400 μl of wash buffer, before addition of 50 μl of TMB solution (Thermo Scientific, cat. # 34028) and incubation at RT for 30 min. The reaction was stopped with stopping solution (Sigma, cat. # S5814), and the absorbance at 450 nm was measured using an ELISA reader (Bio-Rad).

### Cell culture monolayer ECM binding assays

One million cells were plated per well (9.6 cm^2^/well) in a 6-well plate, containing microscope cover slips, and cultured with or without doxycycline for 48 h. Subsequently, supernatant were collected for Western blot analysis, and cell monolayers were washed three times with PBS. After 10-min fixation with 1% paraformaldehyde in PBS, cell monolayers were incubated with a goat anti-human Fc IgG1-cy3-conjugated antibody (1 in 500 in PBS; Jackson Immunoresearch, cat. # 115-165-008) at 4°C overnight. Cover slips were mounted and visualized as described below.

### HUVEC proliferation assay

Primary human umbilical vein endothelial cells (HUVEC-2, BD Biosciences, cat. # 354151) were cultured in 96-well plates (5,000 cells per well) in 100 μl of basic EBM-2 media (Lonza, cat. # CC-3156) per well, along with various amounts of traps (0–218 nM) and VEGF_165_ (1 nM; R&D Systems, cat. # 293-VE-010). After 72 h, 10 μl of AlamarBlue® (Invitrogen, cat. # DAL1025) was added per well and incubated for 18 h. Fluorescence was measured on a plate reader (560EX nm/590EM nm filter settings).

### Heparin column

To test the affinity of recombinant traps to heparin, 1 μg of trap was mixed with 20 μl of immobilized heparin-Sepharose beads (Pierce, cat. # 20207) in 400 μl of 0.05 M phosphate buffer and 0.25 M NaCl and incubated at room temperature for 2 h. Elution was performed with 500 μl of 0.05 M phosphate buffer with NaCl concentrations of 0.25, 0.5, 1.0 and 1.5 and 2.0 M. Ten microlitres from each elution fraction, along with proper controls, was resolved by SDS–PAGE gel, and Western blot was done using a goat anti-human Fc IgG1-HRP-conjugated antibody.

### Pharmacokinetics

To assess the pharmacokinetic profile, traps (4 mg/kg) were injected subcutaneously in 8-week-old male C57BL/6J mice (average 25 g body weight), and blood was collected 1, 2, 4, 6, 24, 48, 96 and 168 h post-injection. The serum levels of the traps were measured using ELISA.

### Xenografts

For the xenograft assays, we used 8- to 12-week-old male nude mice for the PC-3 prostate and A-673 rhabdomyosarcoma cancer lines and 12- to 16-week-old female nude mice for the HT-29 colon cancer line. Briefly, cells were trypsinized and suspended into serum-free media containing 33% Matrigel (BD Biosciences, cat. # 356234). Mice were anesthetized using isoflurane and injected subcutaneously with 5 × 10^6^ transgenic cancer cells or 1 × 10^6^ wild-type cancer cells, per site in both dorsal flanks (150 μl per site). The mice were housed a specific-pathogen-free (SPF) condition, and the care of the animals was in accordance with institutional guidelines (Toronto Centre for Phenogenomics, Toronto, ON, Canada). Doxycycline was administered using doxycycline-containing pellets (0.625 g/kg; Harlan Laboratories, cat. # TD.01306). EGFP expression was monitored using a 388-nm wavelength (blue) LED light source, the appropriate cut-off filters and a conventional camera. Tumour size was monitored using callipers and the volume was calculated using the formula V = (*L* × *W* × *H*)π/6.

### Wound healing assay

Wounding was performed and healing assessed as previously described (Niethammer *et al*, 2002; Seavey *et al*, 2009). Wounds were created in the dorsal area, close to the neck, of male nude mice (8–12 weeks of age) bearing bilateral subcutaneous tumours in the flank areas. For these studies, we used the A-673 cell lines stably transfected with traps. Once the combined size of both tumours was approximately 1.5 cm^3^, doxycycline was administered for 8 days in order to induce the expression of traps. At day 8, mice were anesthetized using isoflurane, and full-thickness wounds were created using 6-mm sterile Biopsy Punch (Miltex, cat. # MIL33-36) at the nape area. One drop of liquid bandage was applied to the wound. Post-operatively, liquid Metacam (meloxicam) was given subcutaneously for 3 days for pain management, and Novo-Trimel (trimethoprim and sulfamethoxazole) was given *per os* for 5 days in order to avoid any bacterial infections. The wound was photographed every other day for a period of 12–14 days. Blood samples were also collected before doxycycline administration, at day 8 and at the end of the study, as described below. At the end of the study (day 12–14), the mice were euthanized and the wound area was dissected and further analysed using haematoxylin and eosin (H&E) staining.

### Plasma collection

Blood samples were collected in Microtainer plasma-separating tubes (Becton Dickinson, cat. # 365985) from retro-orbital sinus during the study and by cardiac puncture of mice under anaesthesia with isoflurane at the end of the study. Samples were centrifuged, aliquoted and stored at −70°C until assayed. All capillary tubing, syringes and needles used for bleeding were heparin-coated to avoid clotting.

### Hypoxia assessment

Tumour hypoxia was detected using a Hypoxyprobe Plus Kit (Natural Pharmacia International Inc.) following the manufacturer's instructions. Briefly, 60 mg/kg of pimonidazole hydrochloride was injected i.p. into tumour-bearing mice 1.5 h before tissues were harvested. Pimonidazole adducts were detected as described below.

### Immunostaining

Blood vessels were detected by staining for endothelial cells using hamster anti-CD31 (PECAM) monoclonal antibody (1:200; Millipore, cat. # MAB1398Z). Vascular basements were examined with rabbit polyclonal antibody against type-IV collagen (1:5000; Cosmo Bio CO, cat. # LSL-LB-1403-EX). Pimonidazole adducts were detected using hypoxyprobe rabbit antisera (1:50; Natural Pharmacia International Inc., cat. # PAB2627). Proliferation was assessed by phospho-histone H3 (PH3) antibody (1:200; Upstate, cat. # 06-570). Secondary antibodies were anti-hamster Cy-3 conjugated for CD31 (1:500), anti-rabbit Cy-5 conjugated for collagen, PH3 and pimonidazole (1:500).

Tumour samples were frozen using O.C.T. compound (Sakura Tissue-Tek, cat. # 4583) immediately after dissection and stored at −70°C. Cryostat sections were cut into fragments of 18 μm thickness and stored at −70°C until assayed. For immunostaining, sections were fixed with cold methanol for 10 min at RT and left to dry. Tracheas were fixed with 1% paraformaldehyde in PBS for 1 h at RT. Specimens were incubated in 5% goat serum in PBS containing 0.3% Triton X-100 (PBS-T) for 1 h at RT in order to permeabilize them and block any non-specific antibody binding. Primary antibodies were incubated at 4°C overnight, and secondary antibodies for 2 h at RT.

### Image acquisition and quantification

Sections were visualized using Zeiss LSM510 Meta two-photon microscope. Images were captured with a Zeiss Axiocam camera connected to the microscope using AxionVision software. Images of at least six fields were taken for each tumour section (from at least three different tumours), while images of 8–12 fields were taken for each trachea specimen. Images were analysed and quantified using ImageJ (http://rsbweb.nih.gov/ij/).

### Histopathology

Tumour samples were fixed in 10% neutral-buffered formalin prior to paraffin embedding and subsequent H&E staining. Digital slides were created from microscope slides using ScanScope CS, and images were acquired using ImageScope viewing software (Aperio Technologies, Inc.).

### Intravitreal injections

Injections were performed into 6- to 8-week-old adult mice (CD1) using a 34-gauge beveled needle attached to the Nanofil submicrolitre injection system (World Precision Instruments, Sarasota, FL, USA) as previously described (Ballios *et al*, 2010). Injections were made into the vitreous fluid by injecting through the sclera 1–2 mm below the limbus. Procedures were performed using a Möller Hi-R 900C surgical microscope (Innova Medical Ophthalmics, Inc., Toronto, ON, Canada).

### Oxygen-induced retinopathy mouse model

Oxygen-induced retinopathy experiments were performed as previously described (Smith *et al*, 1994). Briefly, P7 pups were exposed to hyperoxia (75% oxygen) for 5 days in an enclosed chamber. P12 pups were then returned to room air (∼20% oxygen) and injected with traps (2.5 μg) or saline, and the retinas were examined at either P17 or P21 stages. Injections were performed by injecting 0.5 μl of each reagent using a 33-gauge Hamilton syringe. Mouse eyes were enucleated and flat mounts were performed as previously described (Dorrell *et al*, 2002). The eyes were immunolabelled with GS Lectin (Life Invitrogen, cat. # I32450) overnight and washed vigorously before mounting in antifade media. Images were captured using a Zeiss 700 confocal microscope, and the images were processed with ImageJ (NIH) and Adobe Photoshop software (Adobe). The effects were quantified using published protocols as previously described to quantify the volume of the neovascular tufts and vaso-obliteration (Banin *et al*, 2006).

### ERG measurements and electron microscopy

Electroretinography measurements and electron microscopy were performed as previously described (Dorrell *et al*, 2009).

### Quantification of vascular permeability

Vascular permeability was determined using slightly modified published protocols (Scheppke *et al*, 2008). P12 OIR pups were injected intravitreally with VEGF-traps or PBS. P17 OIR mouse pups were injected (intramuscular) with 100 μl Evans Blue dye (Sigma, cat. # E2129). The eyes were enucleated 4 h later and the retinas were isolated. Several μl of blood was extracted using cardiac punction. Evans Blue dye that accumulated in the retinas was extracted and solubilized in formamide (0.2 ml per retina) at 78°C overnight. The solutions were ultracentrifuged at 4°C at 128,000 g for 45 min to pellet cellular debris. Evans Blue dye in the supernatant was detected with a spectrophotometer at 620 nm (blue signal) and 740 nm (for background subtraction). Non-solubilized blood samples were also spun for 15 min at 3,550 g at 25°C and diluted 1:1,000 for spectrometry. Leakage was calculated with the following equation: [Evans Blue concentration (mg/ml)]/[blood Evans Blue concentration (mg/ml) × circulation time (h)].

### Statistical analysis

Results are reported as mean ± s.e.m. Statistical significance of differences was assessed by one-way ANOVA followed by the Newman–Keul test, using PRISM, ver. 5.0 (Graph-Pad, San Diego, CA, USA).

## The paper explained

### Problem

Excessive production of vascular endothelial growth factor (VEGF-A) leads to abnormal retinal angiogenesis in patients with DR and in newborns suffering of ROP. If left untreated, both conditions often lead to blindness. Current clinical therapies aimed at inhibiting VEGF have shown some success. However, systemic VEGF inhibition has detrimental side effects, such as kidney toxicity, impaired wound healing, highly elevated blood pressure, gastrointestinal perforation, haemorrhage, thrombosis, reversible posterior leukoencephalopathy, cardiac impairment and endocrine dysfunction. Infants—in which organogenesis is still active—are even more critically affected.

## Results

In this study, we developed a novel inhibitor of VEGF, referred to as VEGF Sticky-trap. It comprised of three components: (a) the VEGF-trap region (b) a modified Fc region that ensures short serum half-life and (c) a HBD that allows Sticky-trap to remain at the place of injection. Using a mouse model of DR and ROP, we proved Sticky-trap to be highly effective. In contrast to currently available treatments, once injected intravitreally, Sticky-trap does not exit into the circulation. Instead, it binds the ECM and remains in the eye for a prolonged period of time. Based on these findings, we investigated and showed that local Sticky-trap treatments do not affect distant wound healing, neither do they compromise the kidney vasculature.

## Impact

This is the first report of a local acting angiogenesis inhibitor that can immediately be translated into the clinic as an effective, and safe treatment of DR and ROP. This is of particular importance for elderly patients and pre-mature infants requiring VEGF suppression and who are especially sensitive to potential side effects of such therapies.
