# Supplementary material for: Local acting Sticky-trap inhibits vascular endothelial growth factor dependent pathological angiogenesis in the eye
Source: EMBO Mol Med. 2014 Apr 4;6(5):604–23. doi: 10.1002/emmm.201303708 (PMC4023884; doi:10.1002/emmm.201303708)
Supplement: Supplementary file 1 [file emmm0006-0604-sd1.pdf]

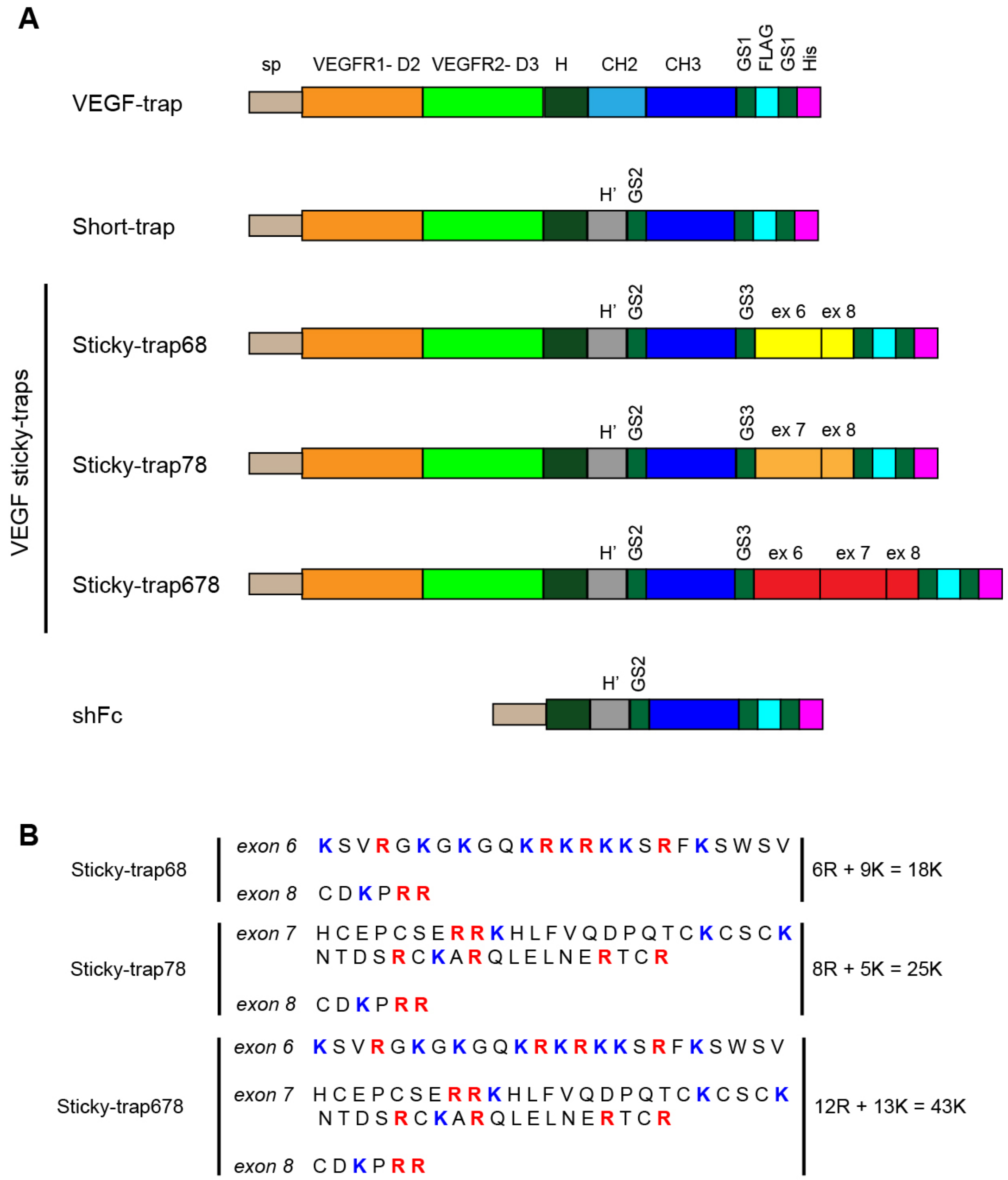

Supplementary Figure 1: (A) Gene structure of traps. ***sp***; signal peptide, ***VEGFR1-D2***; domain-2 of human VEGFR-1, ***VEGFR2-D3***; domain-3 of human VEGFR-2, ***ex 6***, ***ex 7***, and ***ex 8***; mouse VEGF-A exons 6, 7 and 8, respectively, ***H***; hinge domain of IgG1, ***CH2***; heavy chain constant domain-2 of IgG1, ***CH3***; heavy chain constant domain-3 of IgG1, ***H'***; alternative hinge domain (EPKSCDTPPPCPRCPAR **(28)**), ***FLAG*** and ***His***; epitope tags (DYKDDDDK and HHHHHHHH, respectively), ***GS1***, ***GS2***, and ***GS3***; linkers (GGGS, GGGSSGGGS, and GGGAS, respectively). (**B**) Sequence of exons encoding amino acids with affinity for heparan sulphate proteoglycans (HSPs). Amino acid sequence of exons 6, 7 and 8 of mouse VEGF-A are shown and basic amino acids, Arg (R) and Lys (K), are highlighted. The "strength" of each combination is also shown as a numeric value of Lys (K).
