# Supplementary material for: Local acting Sticky-trap inhibits vascular endothelial growth factor dependent pathological angiogenesis in the eye
Source: EMBO Mol Med. 2014 Apr 4;6(5):604–23. doi: 10.1002/emmm.201303708 (PMC4023884; doi:10.1002/emmm.201303708)
Supplement: Supplementary file 2 [file emmm0006-0604-sd2.pdf]

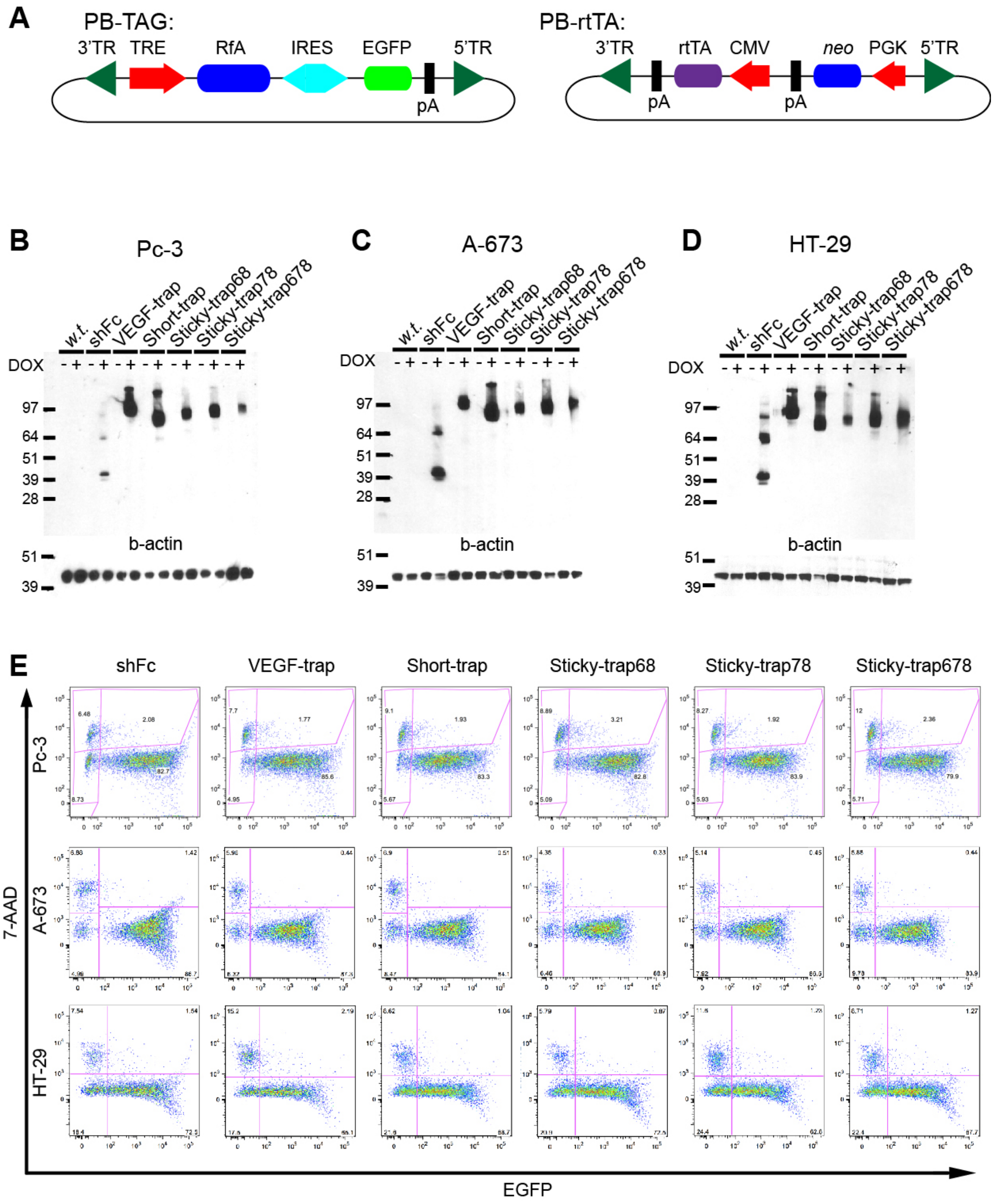

Supplementary Figure 2: (**A**) *piggy*Bac transposons. PB-TAG: tetracycline-regulated promoter (TRE) controlling the expression of the bicistronic transgene of the gene of interest (GOI) with EGFP. The GOI is inserted into the Gateway cassette with reading frame A (RfA). PB-rtTA includes the reverse tetracycline transactivator (rtTA) and the neomycin-resistance gene (neo), driven by CMV and PGK promoter, respectively. (**B**, **C** and **D**). Transgene expression monitored by western blot analysis using an anti-human FcIgG1-HRP antibody. (**E**) Flow cytometry analysis of the expression of EGFP from cancer stable cell lines (Pc-3, A-673 and HT-29) after induction with doxycycline. EGFP expression was analyzed 48 hrs after doxycycline addition to the media. Also see **Supplementary Table I**.
