# Supplementary material for: Local acting Sticky-trap inhibits vascular endothelial growth factor dependent pathological angiogenesis in the eye
Source: EMBO Mol Med. 2014 Apr 4;6(5):604–23. doi: 10.1002/emmm.201303708 (PMC4023884; doi:10.1002/emmm.201303708)
Supplement: Supplementary file 3 [file emmm0006-0604-sd3.pdf]

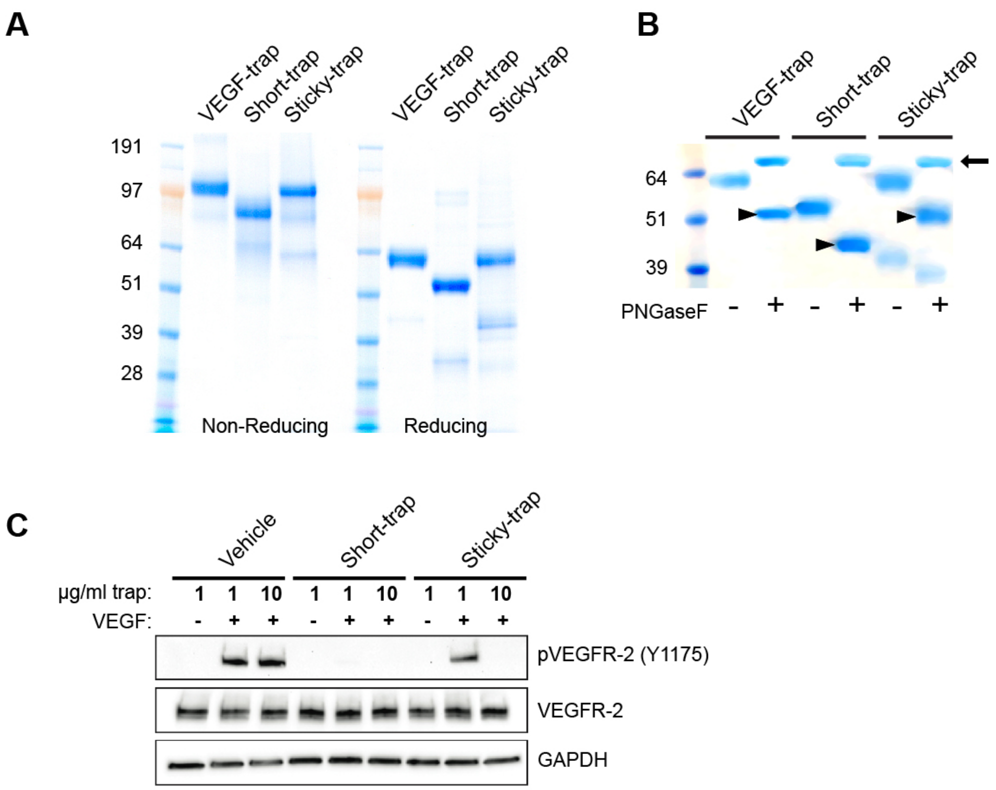

Supplementary Figure 3: (**A**) Reduced and non-reduced SDS-PAGE analysis of recombinant traps (5 µg), stained with Coomassie blue. Molecular weight (kDa) is shown. (**B**) Glycosylation analysis of traps. Two microgram of recombinant proteins were incubated with PNGaseF and separated under reducing conditions by SDS-PAGE, followed by staining with Coomassie blue. Arrowheads indicate the deglycosylated form of traps. Arrow shows recombinant PNGaseF. (**C**) VEGFR-2 tyrosine phosphorylation assay. HUVEC cells were incubated with VEGF pre-incubated with different ratios of traps. VEGFR-2 Y1175 was evaluated by immunoblot analysis.
