# Supplementary material for: Local acting Sticky-trap inhibits vascular endothelial growth factor dependent pathological angiogenesis in the eye
Source: EMBO Mol Med. 2014 Apr 4;6(5):604–23. doi: 10.1002/emmm.201303708 (PMC4023884; doi:10.1002/emmm.201303708)
Supplement: Supplementary file 4 [file emmm0006-0604-sd4.pdf]

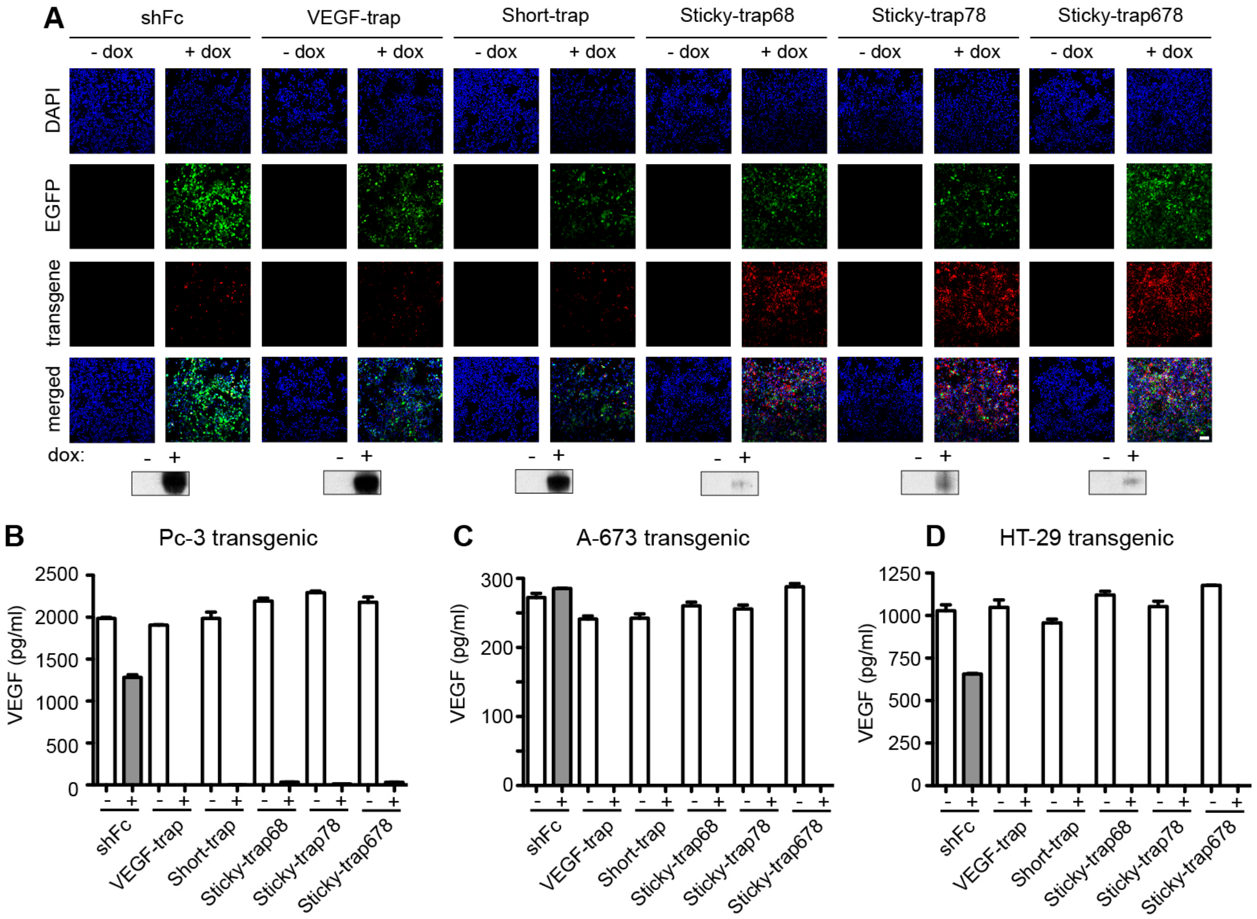

Supplementary Figure 4: (**A**) Immunostaining (red signal) and western blot analysis (on the bottom of image) of traps in A-673 cell monolayers and conditioned supernatant, respectively. A-673 stably transfected lines were cultured with or without doxycycline for 48 hrs. Scale bar, 100 µm. (**B**, **C** and **D**) Free VEGF levels in the conditioned of Pc-3, A-673 and HT-29 cancer transgenic cell lines. Media was collected after 48 hrs of culture with or without the addition of doxycycline.
