# Supplementary material for: Local acting Sticky-trap inhibits vascular endothelial growth factor dependent pathological angiogenesis in the eye
Source: EMBO Mol Med. 2014 Apr 4;6(5):604–23. doi: 10.1002/emmm.201303708 (PMC4023884; doi:10.1002/emmm.201303708)
Supplement: Supplementary file 5 [file emmm0006-0604-sd5.pdf]

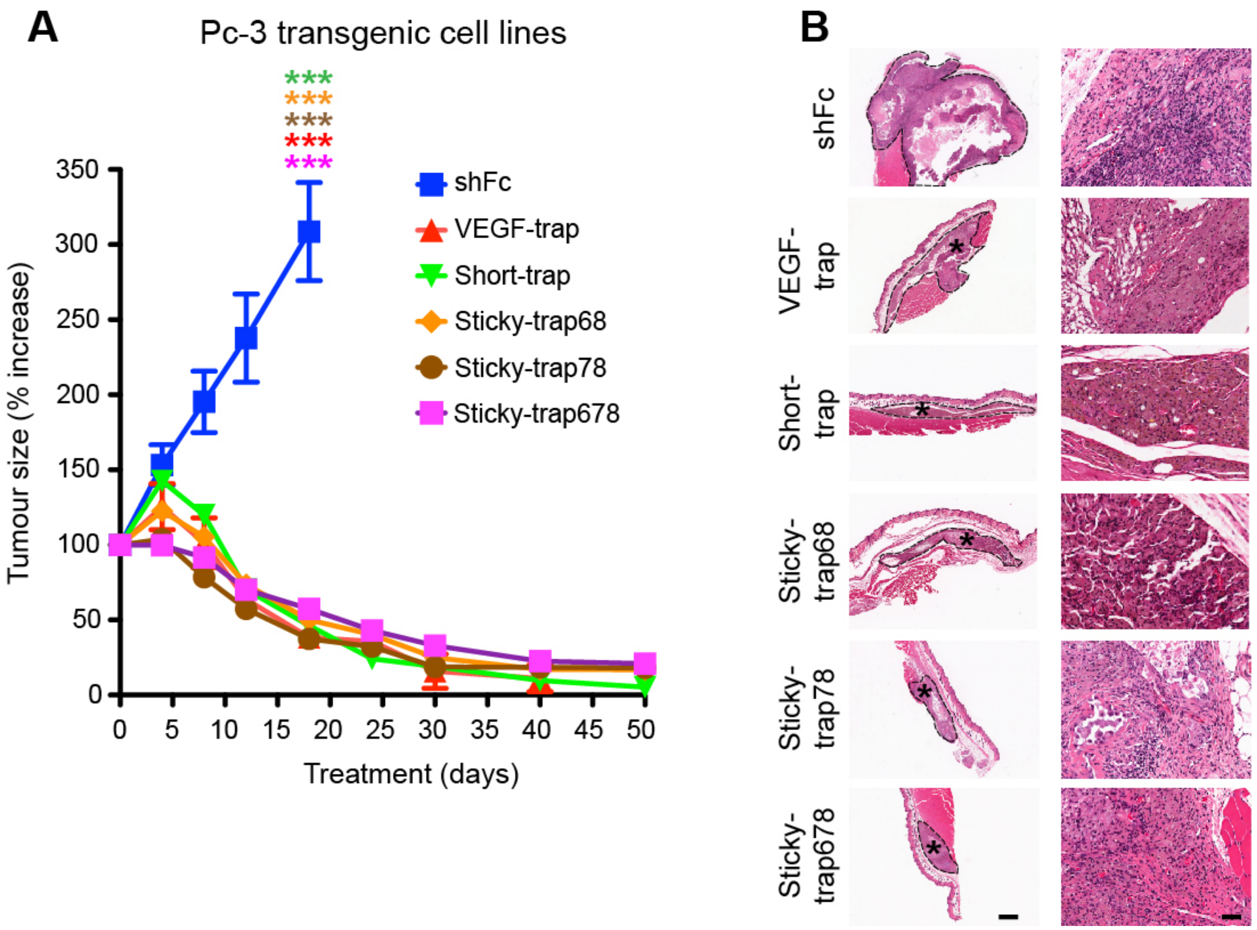

Supplementary Figure 5: (**A**) Tumour growth kinetics of xenografts established with Pc-3 transgenic cancer cells. Error bars represent s.e.m. (n=10-12 tumours; ***P<0.001, one-way ANOVA). (**B**) H&E analysis of Pc-3 xenograft sections. Asterisks indicate small tumour nodules under the skin 50 days after initiation of transgene (traps) expression. Scale bars, 500 µm (left column) and 25 µm (right column).
