# Supplementary material for: Local acting Sticky-trap inhibits vascular endothelial growth factor dependent pathological angiogenesis in the eye
Source: EMBO Mol Med. 2014 Apr 4;6(5):604–23. doi: 10.1002/emmm.201303708 (PMC4023884; doi:10.1002/emmm.201303708)
Supplement: Supplementary file 6 [file emmm0006-0604-sd6.pdf]

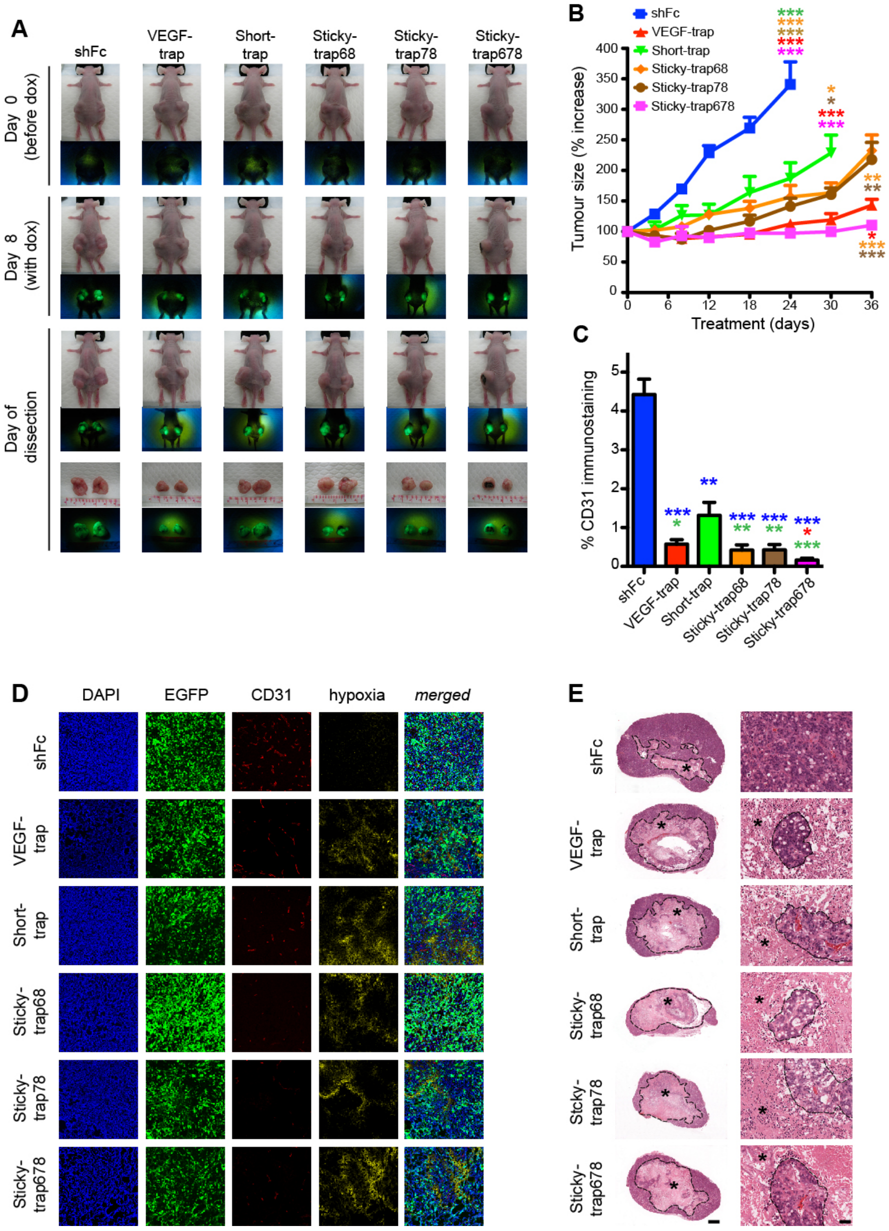

Supplementary Figure 6: HT-29 xenograft studies. (**A**) Bright field and EGFP images of HT-29 transgenic line xenografts. No EGFP was detected before feeding the tumour-bearing mice with *dox*-chow (Day 0). Exposure to doxycycline resulted in high EGFP expression levels at "day 8", which was maintained until the animals were euthanized, "day of dissection." Dissected tumours are also shown. (**B**) Tumour growth rate of xenografts established with transgenic cancer cell lines. Transgene (*i.e.* trap) induction by administration of doxycycline-containing chow, once the tumours reached an average size of 500 mm^3. Error bars represent s.e.m. (n=12; ***P<0.001, ***P<0.01, *P<0.05, one-way ANOVA). (**C**) Microvascular density. Error bars represent s.e.m. (n=8-10; ***P<0.001, one-way ANOVA) (**D**) Confocal images of xenograft frozen sections immunostained for vessels (anti-CD31 antibody) and hypoxia (pimonidazole rabbit antisera). Green: EGFP expression from xenografts. Blue: DAPI. Scale bar, 100 µm. (**E**) H&E analysis of xenograft sections. Purple: viable tumour nuclei. Pink: necrotic regions (asterisks). Scale bars, 500 µm (left column) and 25 µm (right column).
