# Supplementary material for: Local acting Sticky-trap inhibits vascular endothelial growth factor dependent pathological angiogenesis in the eye
Source: EMBO Mol Med. 2014 Apr 4;6(5):604–23. doi: 10.1002/emmm.201303708 (PMC4023884; doi:10.1002/emmm.201303708)
Supplement: Supplementary file 7 [file emmm0006-0604-sd7.pdf]

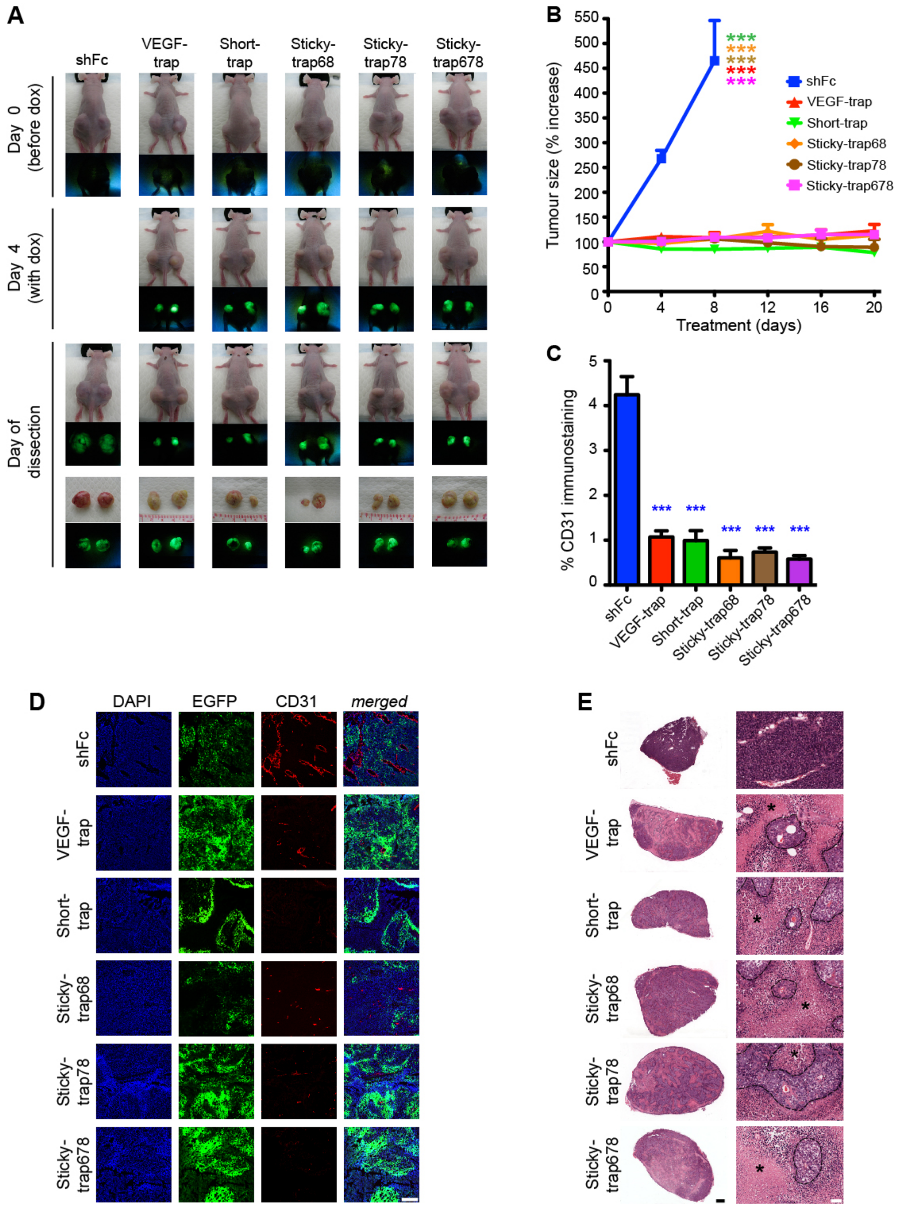

Supplementary Figure 7: A-673 xenograft studies. (**A**) Bright field and EGFP images of A-673 transgenic line xenografts. No EGFP was detected before *dox*-induction (Day 0). Exposure to doxycycline resulted in high EGFP expression levels at "day 4", and maintained until the animals were euthanized, "day of dissection." Dissected tumours: red colour (shFc group) marks blood vessels. (**B**) Tumour growth rate of xenografts established with transgenic cancer cell lines. Transgene (*i.e.* trap) induction by administration of doxycycline-containing chow, once the tumours reached an average size of 500 mm^3. Error bars represent s.e.m. (n=12; ***P<0.001, ***P<0.01, *P<0.05, one-way ANOVA). (**C**) Microvascular density. Error bars represent s.e.m. (n=11-13; ***P<0.001, one-way ANOVA). (**D**) Confocal images of xenograft frozen sections immunostained for vessels using an anti-CD31 antibody. Green: EGFP expression from the xenografts. Blue: DAPI. Scale bar, 100 µm. (**E**) H&E analysis of xenograft sections. Purple: viable tumour nuclei. Pink: necrotic regions (asterisks). Scale bars, 500 µm (left column) and 25 µm (right column).
