# Supplementary material for: Local acting Sticky-trap inhibits vascular endothelial growth factor dependent pathological angiogenesis in the eye
Source: EMBO Mol Med. 2014 Apr 4;6(5):604–23. doi: 10.1002/emmm.201303708 (PMC4023884; doi:10.1002/emmm.201303708)
Supplement: Supplementary file 8 [file emmm0006-0604-sd8.pdf]

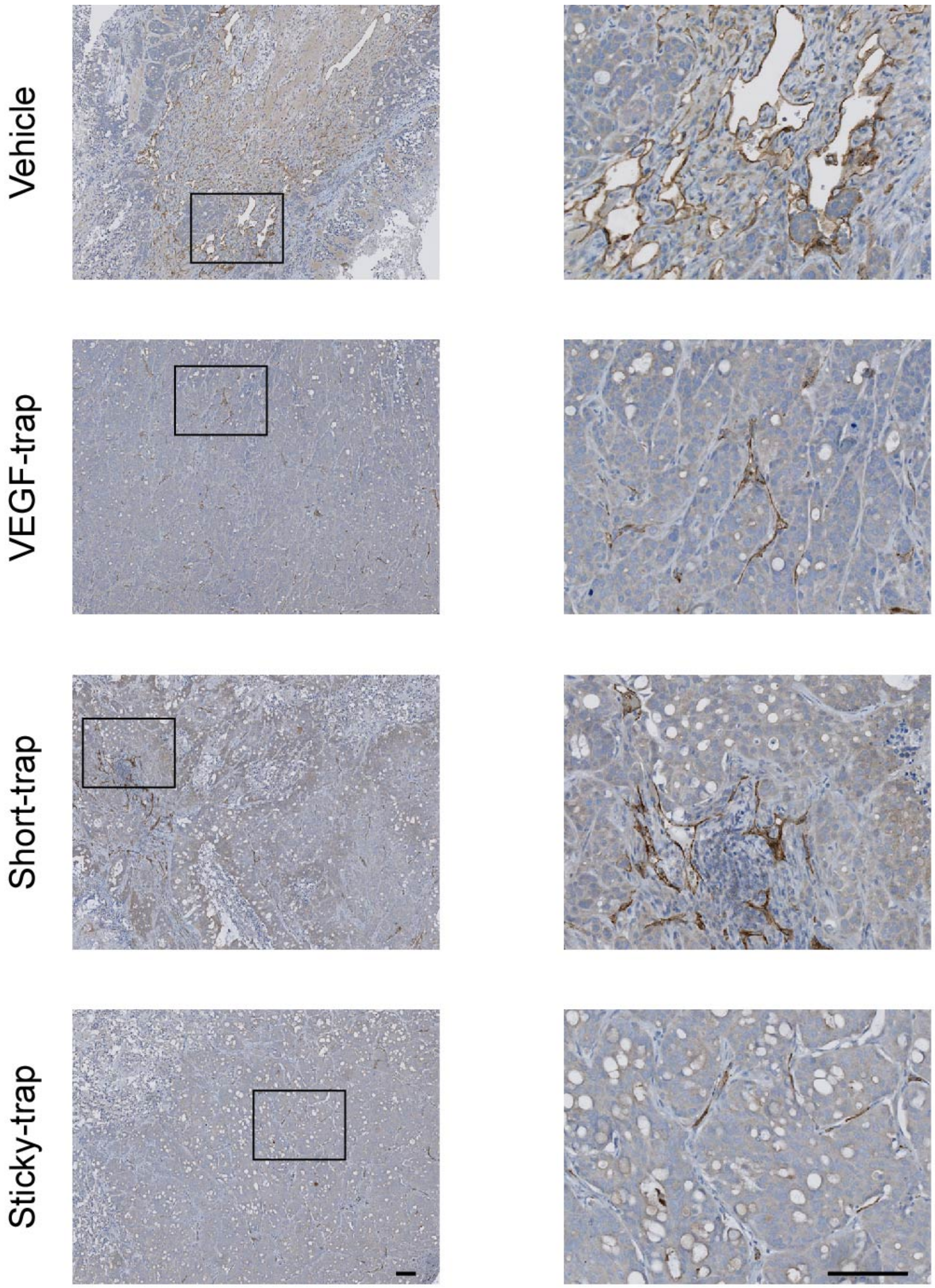

Supplementary Figure 8: Paraffin sections of HT-29 xenografts, treated with traps (**Figure 3A**), stained for vessels using anti-CD31 antibody. Scale bars, 100 µm.
