# Supplementary material for: Local acting Sticky-trap inhibits vascular endothelial growth factor dependent pathological angiogenesis in the eye
Source: EMBO Mol Med. 2014 Apr 4;6(5):604–23. doi: 10.1002/emmm.201303708 (PMC4023884; doi:10.1002/emmm.201303708)
Supplement: Supplementary file 11 [file emmm0006-0604-sd11.pdf]

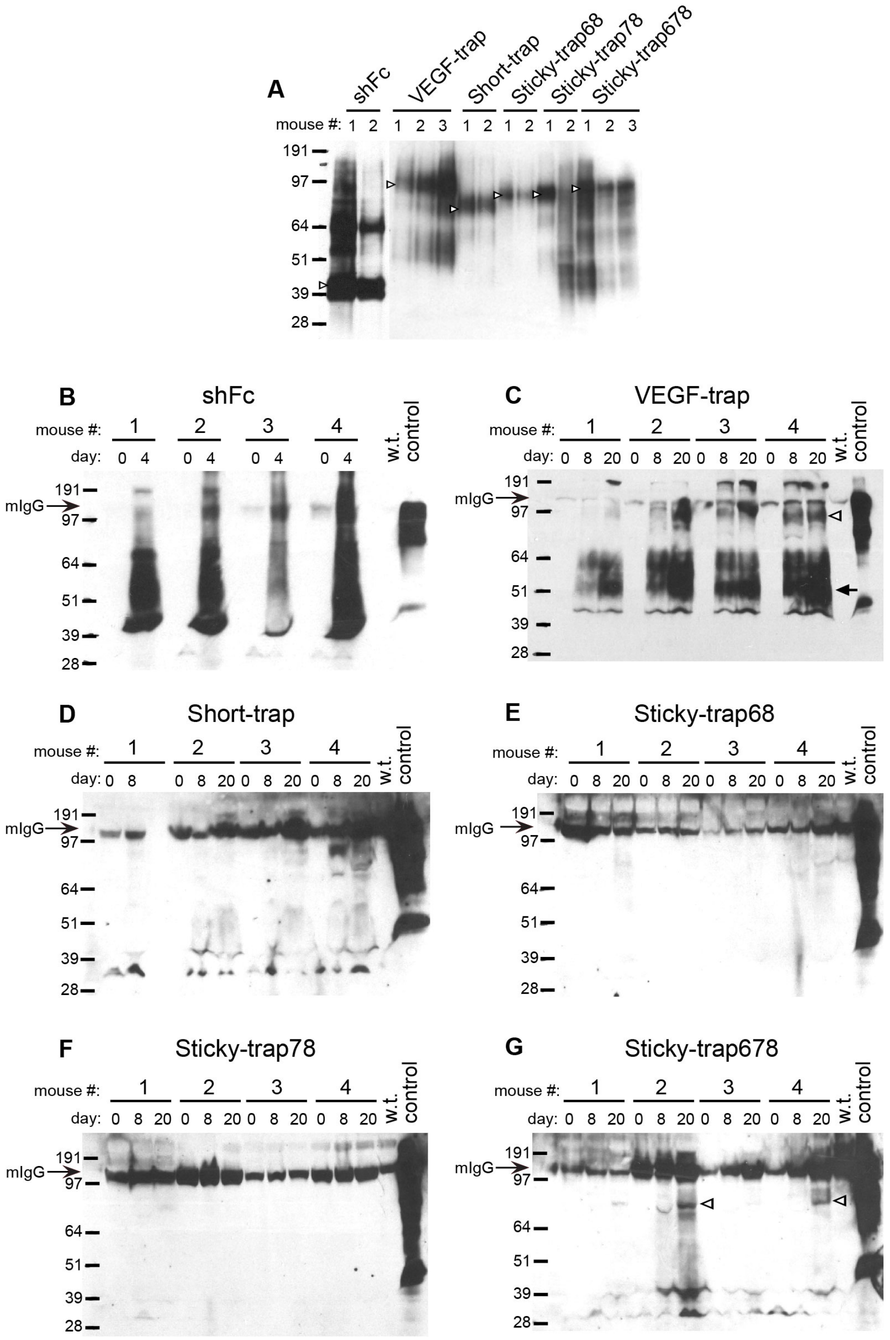

Supplementary Figure 11: Western blot analysis of transgene expression in A-673 tumours (**A**), and traps circulation (**B**-**G**). For western blot analysis 2 µl of serum were loaded for the VEGF-trap group and 10 µl for the rest. Serum from non-tumour bearing animals fed with *dox*-chow were used as "w.t." controls. The tumour extracts and serum samples are from the matching samples. Recombinant hIgG was used as western blot control. Mouse IgG was also detectable by the anti-human IgG1-HRP antibody (arrow).
