# Supplementary material for: Local acting Sticky-trap inhibits vascular endothelial growth factor dependent pathological angiogenesis in the eye
Source: EMBO Mol Med. 2014 Apr 4;6(5):604–23. doi: 10.1002/emmm.201303708 (PMC4023884; doi:10.1002/emmm.201303708)
Supplement: Supplementary file 12 [file emmm0006-0604-sd12.pdf]

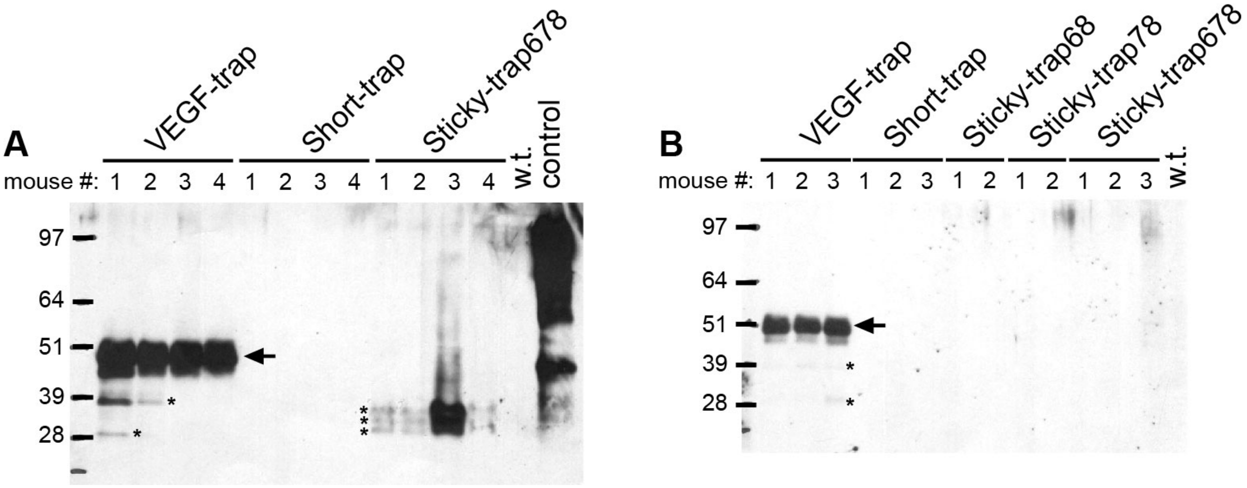

Supplementary Figure 12: Detection of traps in urine samples of tumour bearing mice. Urine was collected 20 and 24 days after transgene induction from A-673 (**A**) and HT-29 (**B**) tumour bearing mice. Arrows indicate intact traps, while asterisks degraded forms. Two microliters of urine was used from each sample for western blot analysis, and traps were detected using anti-human IgG1-HRP antibody. Recombinant hIgG was used as control.
