# Supplementary material for: Local acting Sticky-trap inhibits vascular endothelial growth factor dependent pathological angiogenesis in the eye
Source: EMBO Mol Med. 2014 Apr 4;6(5):604–23. doi: 10.1002/emmm.201303708 (PMC4023884; doi:10.1002/emmm.201303708)
Supplement: Supplementary file 13 [file emmm0006-0604-sd13.pdf]

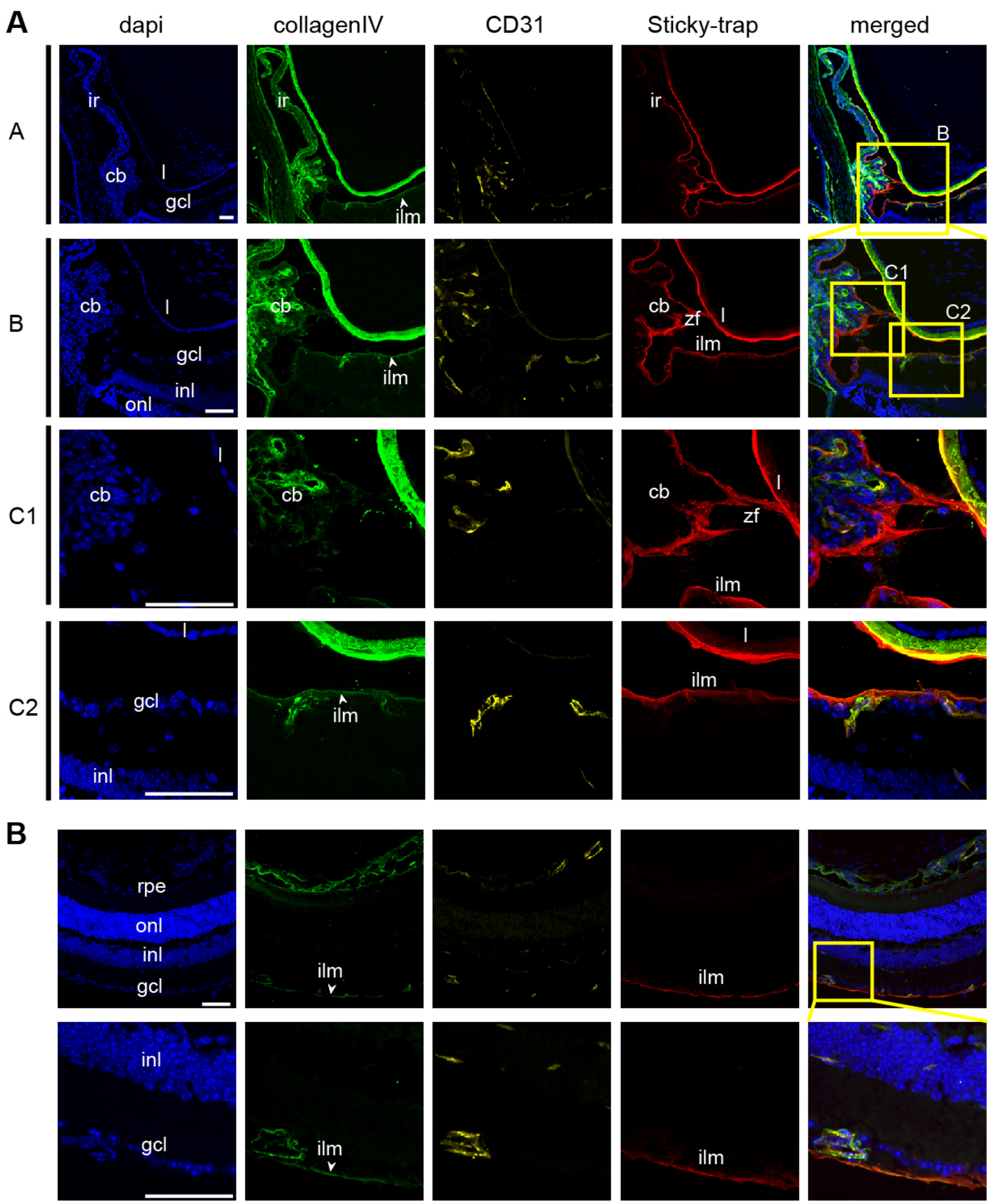

Supplementary Figure 13: Immunostaining analysis of mouse eye cross sections for Sticky-trap. Dissections were performed 2 days post intravitreal injection of Sticky-trap (10 µg). Sticky-trap (red signal) binds to the iris (**A**; panel A), ciliary body (**A**; panels B, C1), zonular fibers (**A**; panels B, C1), lens (**A**; panels B, C1, C2), and inner-limiting membrane (**A**; panels B, C1, C2, and **B**) of the eye. *ir*; iris, cb; ciliary body, *l*; lens, *ilm*; inner limiting membrane, *inl*; inner nuclear membrane, *onl*; outer nuclear membrane, *gl*; ganglion cell layer, *rpe*; retinal pigmented epithelium cell layer. Scale bars, 50 µm.
