# Supplementary material for: Local acting Sticky-trap inhibits vascular endothelial growth factor dependent pathological angiogenesis in the eye
Source: EMBO Mol Med. 2014 Apr 4;6(5):604–23. doi: 10.1002/emmm.201303708 (PMC4023884; doi:10.1002/emmm.201303708)
Supplement: Supplementary file 14 [file emmm0006-0604-sd14.pdf]

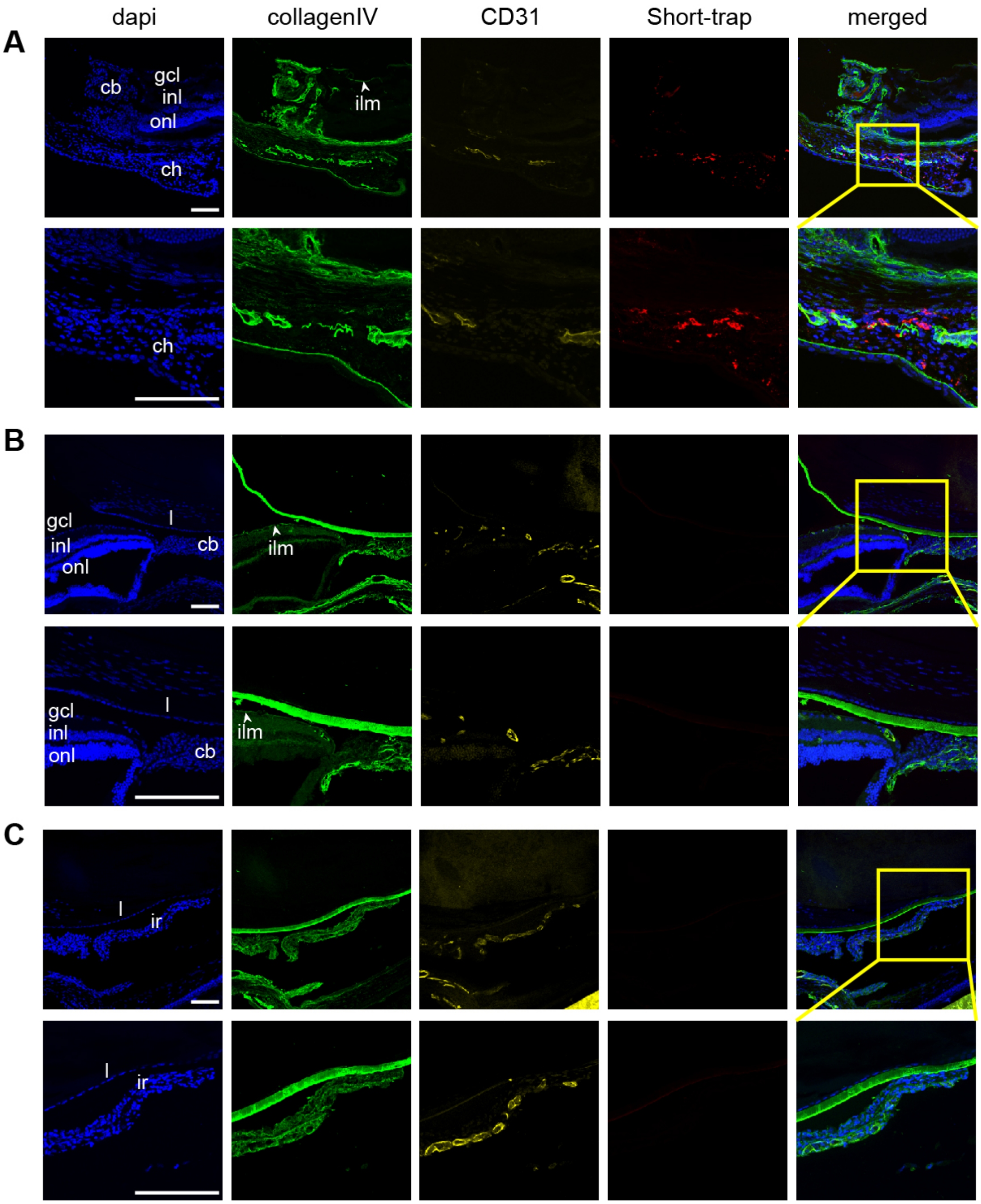

Supplementary Figure 14: Immunostaining of mouse eye cross sections for Short-trap. Dissections were performed 2 hrs post intravitreal injection of Short-trap (10 µg). Minute amounts of trap were detected in the choroid (**A**), while they were not detectable in other areas of the eye such as the inner limiting membrane, and ciliary body (**B**); and the iris, and lens (**C**). *ir*; iris, cb; ciliary body, *l*; lens, *ilm*; inner limiting membrane; *inl*; inner nuclear membrane; *onl*; outer nuclear membrane, *gl*; ganglion cell layer, *rpe*; retinal pigmented epithelium cell layer, *ch;* choroid. Scale bars, 100 µm.
