# Supplementary material for: Local acting Sticky-trap inhibits vascular endothelial growth factor dependent pathological angiogenesis in the eye
Source: EMBO Mol Med. 2014 Apr 4;6(5):604–23. doi: 10.1002/emmm.201303708 (PMC4023884; doi:10.1002/emmm.201303708)
Supplement: Supplementary file 16 [file emmm0006-0604-sd16.pdf]

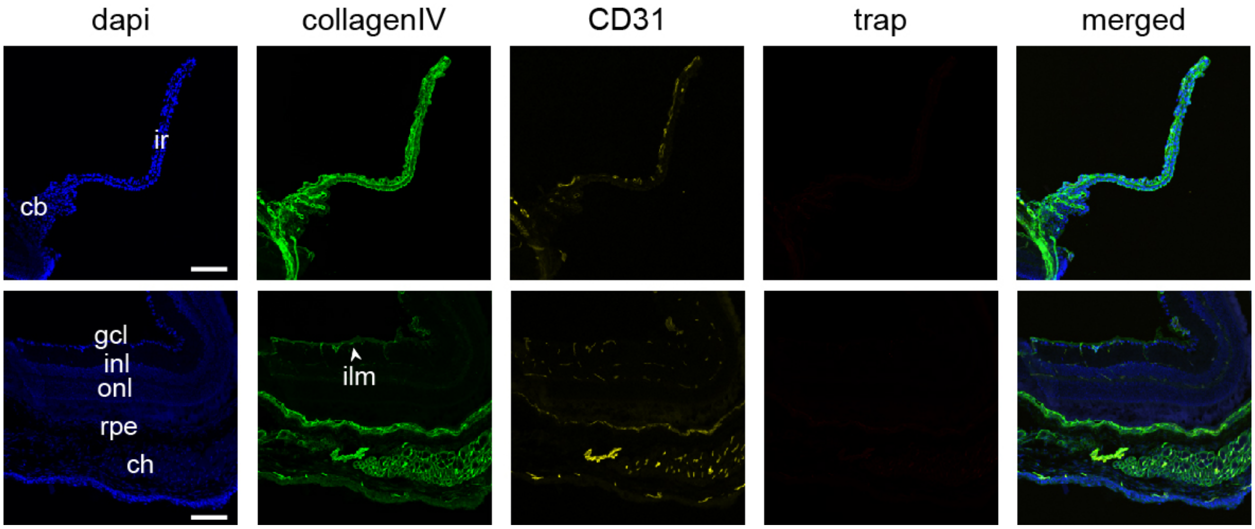

Supplementary Figure 16: Immunostaining analysis of mouse eyes cross sections; dissected 2 hrs post intravitreal injection with PBS. *ir*; iris, cb; ciliary body, *ilm*; inner limiting membrane; *inl*; inner nuclear membrane; *onl*; outer nuclear membrane, *gl*; ganglion cell layer, *rpe*; retinal pigmented epithelium cell layer, *ch;* choroid. Scale bars, 100 µm.
