# Supplementary material for: Local acting Sticky-trap inhibits vascular endothelial growth factor dependent pathological angiogenesis in the eye
Source: EMBO Mol Med. 2014 Apr 4;6(5):604–23. doi: 10.1002/emmm.201303708 (PMC4023884; doi:10.1002/emmm.201303708)
Supplement: Supplementary file 17 [file emmm0006-0604-sd17.pdf]

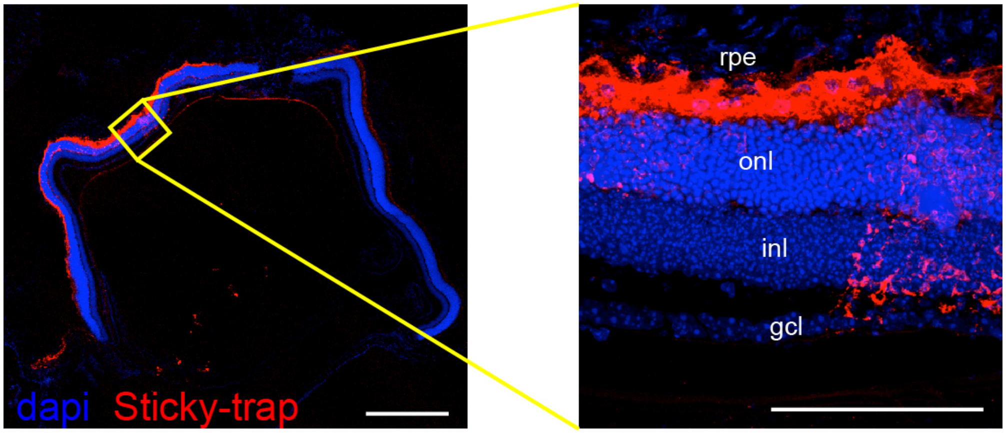

Supplementary Figure 17: Immunostaining analysis of mouse eye cross sections; dissected 3 days post subretinal injection of Sticky-trap (2.5 µg). *inl*; inner nuclear membrane; *onl*; outer nuclear membrane, *gl*; ganglion cell layer, *rpe*; retinal pigmented epithelium cell layer. Scale bars 500 µm (left panel) and 100 µm (right panel).
