# Supplementary material for: Local acting Sticky-trap inhibits vascular endothelial growth factor dependent pathological angiogenesis in the eye
Source: EMBO Mol Med. 2014 Apr 4;6(5):604–23. doi: 10.1002/emmm.201303708 (PMC4023884; doi:10.1002/emmm.201303708)
Supplement: Supplementary file 18 [file emmm0006-0604-sd18.pdf]

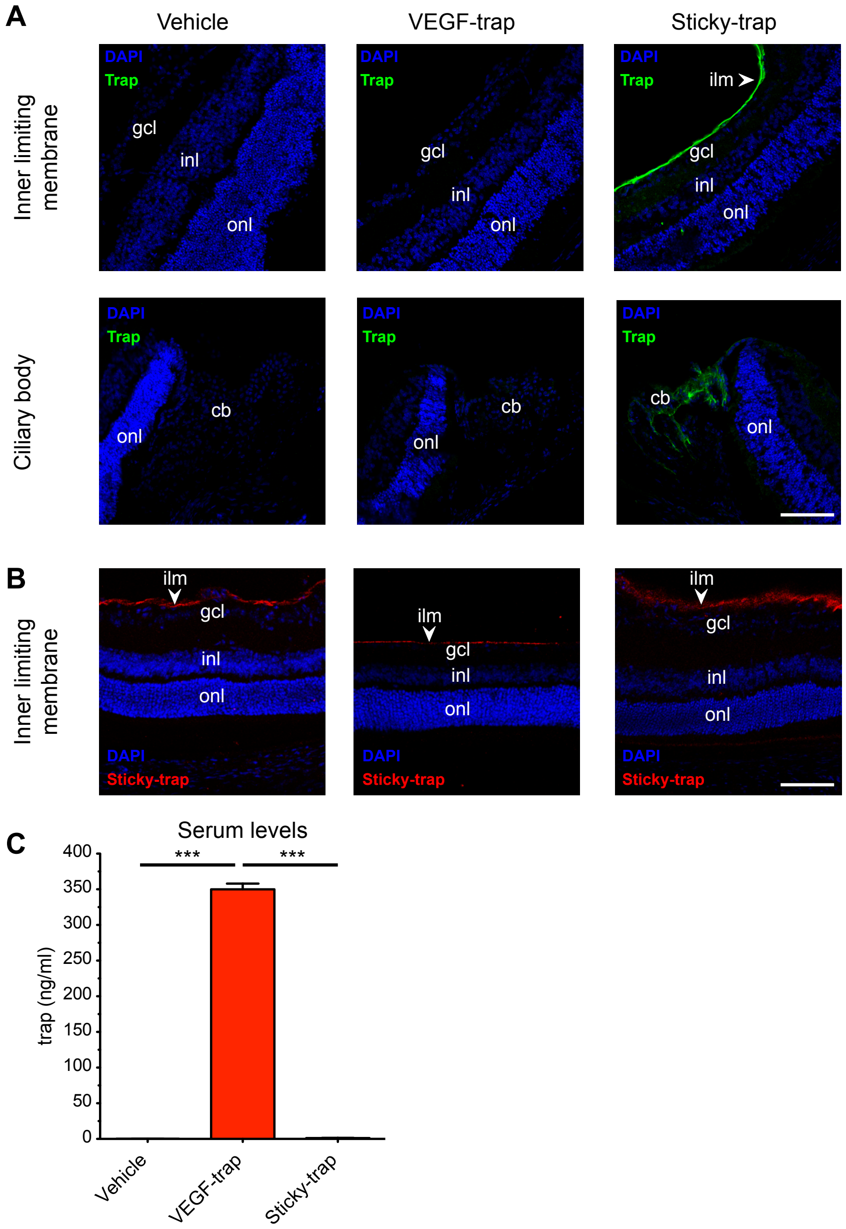

Supplementary Figure 18: Biodistribution analysis of traps in the eyes of the OIR mouse model. Frozen cross sections were immunostained with an anti-human FcIgG1 antibody. (**A**) Traps were injected upon return to normoxic conditions at P12, and the eyes were dissected at P17. (**B**) Traps were injected at P7, before exposure to hyperoxia, and the eyes were dissected at P17. *ilm*; inner limiting membrane, *inl*; inner nuclear membrane, *onl*; outer nuclear membrane, *gcl*; ganglion cell layer. Scale bars, 100 µm. (**C**) Serum levels of traps 5 days post intravitreal injections. Error bars represent s.e.m. (n=8-10; ***P<0.001, one-way ANOVA).
