# Supplementary material for: Local acting Sticky-trap inhibits vascular endothelial growth factor dependent pathological angiogenesis in the eye
Source: EMBO Mol Med. 2014 Apr 4;6(5):604–23. doi: 10.1002/emmm.201303708 (PMC4023884; doi:10.1002/emmm.201303708)
Supplement: Supplementary file 19 [file emmm0006-0604-sd19.pdf]

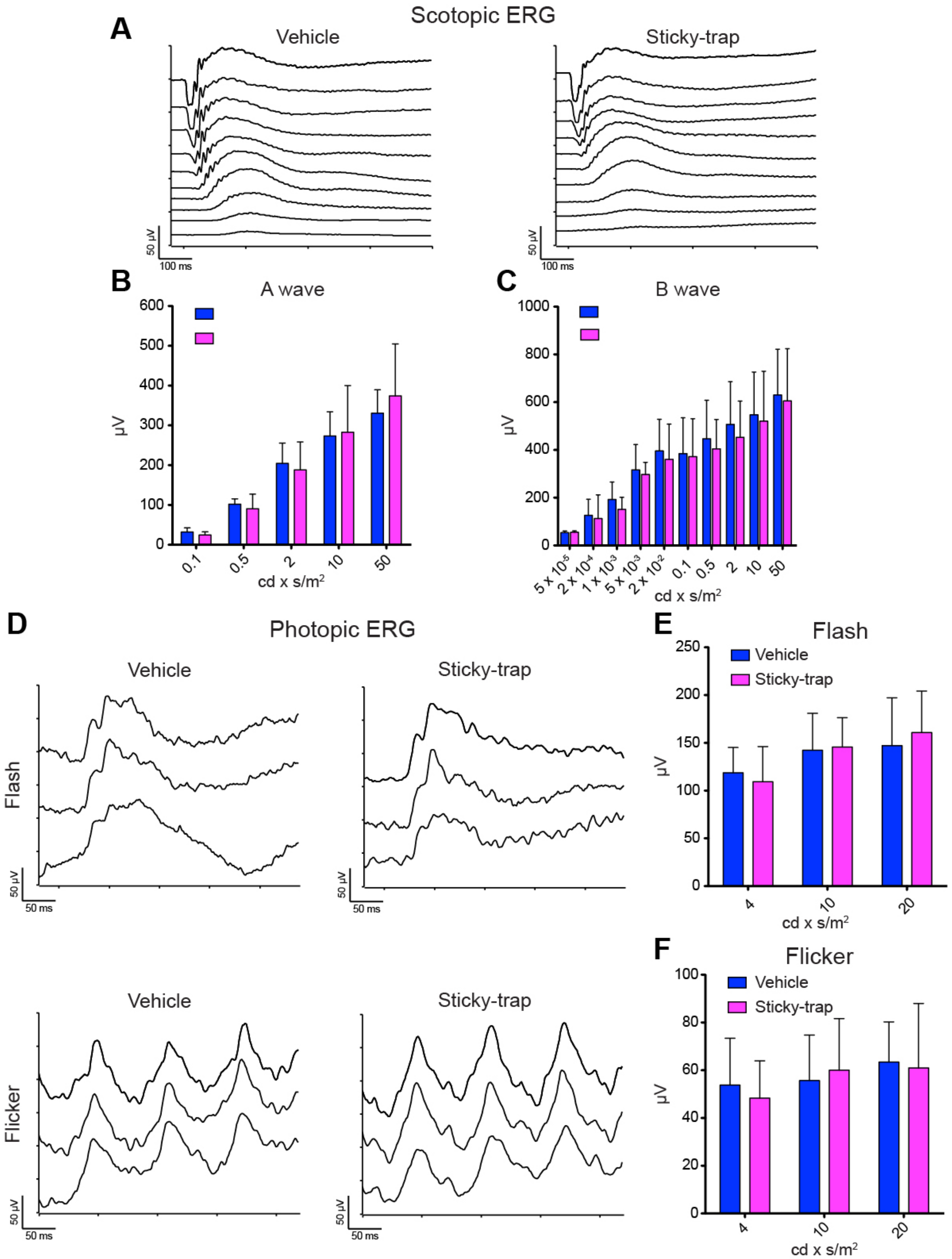

Supplementary Figure 19: Effect of Sticky-trap in vision after single subretinal injection (2.5µg) into the right eye of C57BL/6J. The contralateral eye was injected with vehicle. The function of rod and cone photoreceptors was evaluated with scotopic (**A-C**) and photopic (**D-F**) ERGs, respectively, one week after treatment. No reduction of either A- or B- wave was observed indicating the normal function of rod photoreceptors (**B, C**) (n=8). Similarly, both b-wave amplitudes from flash and first peaks from flicker appeared normal (**E, F**), indicating the normal function of cone photoreceptors (n=8).
