# Supplementary material for: Local acting Sticky-trap inhibits vascular endothelial growth factor dependent pathological angiogenesis in the eye
Source: EMBO Mol Med. 2014 Apr 4;6(5):604–23. doi: 10.1002/emmm.201303708 (PMC4023884; doi:10.1002/emmm.201303708)
Supplement: Supplementary file 20 [file emmm0006-0604-sd20.pdf]

Supplementary Table I

Percentage of cells expressing GFP after 48hrs doxycycline determined by single-cell flow cytometry.

	Pc-3	A-673	HT-29	*Average*
shFc	90	95	80	88.3
VEGF-trap	95	93	79	89
Short-trap	95	91	76	87.3
Sticky-trap68	95	93	78	88.6
Sticky-trap78	95	92	72	86.3
Sticky-trap678	95	91	75	87
Average	94.16	92.5	76.6	

A very high proportion of the resistant cells became double transgenic and responded to doxycycline
