# Supplementary material for: Local acting Sticky-trap inhibits vascular endothelial growth factor dependent pathological angiogenesis in the eye
Source: EMBO Mol Med. 2014 Apr 4;6(5):604–23. doi: 10.1002/emmm.201303708 (PMC4023884; doi:10.1002/emmm.201303708)
Supplement: Supplementary file 21 [file emmm0006-0604-sd21.pdf]

Local acting Sticky-trap inhibits VEGF dependent pathological angiogenesis in the eye

Iacovos P. Michael, Peter D. Westenskow, Sabiha Hacibekiroglu, Alissa Cohen Greenwald, Brian G. Ballios, Toshihide Kurihara, Zhijie Li, Carmen M. Warren, Puzheng Zhang, Edith Aguilar, Laura Donaldson, Valentina Marchetti, Takeshi Baba, Samer M. Hussein, Hoon-Ki Sung, M. Luisa Iruela-Arispe, James M. Rini, Derek van der Kooy, Martin Friedlander, Andras Nagy

Corresponding author: Andras Nagy, Mount Sinai Hospital

Review timeline:

Submission date:	07 December 2013
Editorial Decision:	21 January 2014
Revision received:	06 February 2014
Editorial Decision:	24 February 2014
Revision received:	02 March 2014
Accepted:	05 March 2014

Transaction Report:

(Note: With the exception of the correction of typographical or spelling errors that could be a source of ambiguity, letters and reports are not edited. The original formatting of letters and referee reports may not be reflected in this compilation.)

Editor: Roberto Buccione

1st Editorial Decision 21 January 2014

Thank you for the submission of your manuscript to EMBO Molecular Medicine. We have now received reports from the three Reviewers whom we asked to evaluate your manuscript. We are sorry that it has taken longer than usual to get back to you on your manuscript due to the unexpected delay in obtaining one Reviewer's evaluation.

Hopefully, the long wait will be mitigated by the fact that, as you will see, all three Reviewers are supportive of your work and underline its considerable potential interest. They do, however, collectively express a few concerns that require your attention and action.

Reviewer 1 has one major concern related to the issue of the specific binding of the modified Sticky-Trap and would like to better understand the basis for this selectivity.

Reviewer 3 would like to understand how the Sticky-Trap accesses VEGF in the retinal tissue, and suggests further experimentation to define this. S/he also mentions other important issues, especially in a translational perspective, that require your action and which should not prove difficult to address

While publication of the paper cannot be considered at this stage, we would be pleased to consider a suitably revised submission, with the understanding that the Reviewers' concerns must be fully addressed with additional experimental data where appropriate.

Please note that it is EMBO Molecular Medicine policy to allow a single round of revision only and that, therefore, acceptance or rejection of the manuscript will depend on the completeness of your responses included in the next, final version of the manuscript.

I look forward to receiving your revised manuscript as soon as possible.

***** Reviewer's comments *****

Referee #1 (Comments on Novelty/Model System):

Abnormal vascularisation of the retina for those with wet macular degeneration, diabetic retinopathy and retinopathy of prematurity

There are 2 agents commonly used for retinopathy- VEGF antibody and VEGF Trap made as a recombinant protein from domains of VEGFR-1 and VEGFR-2. The problems with these is that they may leak systemically and have effects in premature babies which can be detrimental. Also they may not localise specifically to abnormal angiogenesis areas in the retina and their short duration of effect requiring injections every month or at best 2 months in adults.

The investigators generated a novel form of VEGF receptor, VEGF Trap, which had sticky domains coding exon 6, 7 and 8 or variants of those of VEGF. These domains of VEGF are responsible for its binding to heparin in extracellular matrix. They used appropriate controls with and without sticky traps as well as the initial VEGF Trap molecule without any of these domains, as clinically used.

The initial test was in tumour angiogenesis with cell lines expressing the various types of traps. This led to the selection of sticky trap 6, 7, 8 and its recombinant expression. Systemic pharmacology was undertaken to show that it was rapidly metabolised, did not localise in normal tissues and did not interfere with wound healing. However, systemic injection could block tumour angiogenesis. It showed minimum toxicity in normal tissues compared to VEGF Trap, particularly investigating function in neonates and on tracheal blood vessels. It then undertook studies with intraocular injection into the vitreous. This showed the sticky trap has much better retention for at least 12 days compared to VEGF Trap, which had disappeared by 2 days.

Sub retinal injections were also given. This again showed retention in the sub retinal space. In comparative studies of the oxygen induced retinopathy the sticky trap was superior in all endpoints and showed no systemic leak. Additionally, it was not toxic to the eye in terms of normal retinal function. Thus, this represents a unique new molecule with exceptional pharmacology properties for local control of abnormal angiogenesis in the eye. This represents a significant step forward in all aspects of safety and enhanced efficacy and therefore meets a major need for dealing with retinopathy of prematurity and probably also substantial benefit for adult eye disease.

I think the work was extremely well performed with appropriate control, including consideration on the potential value in tumour angiogenesis where selective binding to vasculature could increase the safety margin.

Major comment

The one missing issue here, however, is understanding why it binds so specifically around the tumours and the vascular abnormalities of prematurity but not to normal tissues or to wounding or to other areas which are known to have high VEGF for example the glomerulus- so why is there this highly specific binding? What are the binding components, what is the basis for the selectivity; this is not mentioned in the discussion and there is no experimental data on this.

One might a priori have expected it to bind everywhere that VEGF is detected, but it is clearly not the case. I would recommend at least discussion of this and perhaps some initial evidence for binding differentiated extracellular matrix around tumours or from abnormal ocular vascular could be provided.

Referee #2 (Comments on Novelty/Model System):

This is exceptionally thoroughly and convincingly performed study that has obvious clinical potential. I do not have additional requests regarding new experimentation (this has not happened for many years, although I review tens of manuscripts every year).

Referee #2 (Remarks):

This is exceptionally thoroughly and convincingly performed study that has obvious clinical potential.

Referee #3 (Remarks):

This manuscript reports a very exciting study of a novel pharmacological inhibitor VEGF named VEGF Sticky-trap. Compared to traditional VEGF-trap, Sticky-trap shows a similar anti-angiogenic effect and strong capacity to inhibit VEGF signaling. Uniquely, Sticky-trap binds to extracellular matrix through heparin-binding domains and remains in the administration site without entering the circulation. The study demonstrates that local administration of Sticky-trap has little impact on distal wound healing, suggesting that this treatment will have low systemic side-effects. Thus, this novel inhibitor of VEGF may provide an effective and safer treatment for ocular neovascularization and tumor.
Main points:
1. Fig. 1G shows that Sticky-traps suppressed endothelial cell proliferation in a dose-dependent manner, while in VEGF-trap and Short-trap groups, the inhibitory effect reached a plateau at doses of 10 pM and higher. Interestingly, results from Fig. 1F and Suppl. Figs. 3 and 4 suggest that VEGF-trap and Short-trap had similar, if not stronger, effects on VEGF and VEGF-R2 inhibition. What potential mechanisms could lead to Sticky-trap's more potent inhibition on VEGF-induced proliferation?
2. Suppl. Fig. 18: It seems that Sticky-trap does not across the inner limiting membrane after an intravitreal injection. Thus, it is understandable that intravitreal injection of Sticky-trap will not affect retinal function and outer retina integrity (Fig. 9). But would retinal ganglion cells and neurons of the inner retina be affected? Chronic retinal neuronal injury may occur after an injection.
3. In Suppl. Fig. 17, large amount of Sticky-trap were detected in the inner and outer segment of photoreceptors after a subretinal injection. In this case, would Stick-trap cause any adverse effect on photoreceptors and choroidal blood vessels? Would subretinal injection of Stick-trap be sufficient to suppress choroidal neovascularization, a hallmark pathological feature of wet AMD?
4. If Sticky-trap does not across the inner limiting membrane and get into the retina, how could the trap bind to VEGF and inhibit VEGF/VEGF-R function in retinal tissue? Biochemical assays of retinal VEGF levels and retinal VEGF-R activation (tyrosine phosphorylation assay) should be performed to confirm the effects of Sticky-trap on retinal VEGF signaling and to delineate whether the anti-angiogenic and anti-permeability effects of Sticky-trap is through inhibition of retinal VEGF system.
5. Would the binding of Sticky-trap to ECM affect the pharmacokinetics and anti-VEGF efficacy in the treatment of retinal/choroidal neovascularization? Please discuss.
6. In some metastatic tumors, local injection may be difficult to apply. Potential implication of Sticky-trap in the treatment of these diseases should be discussed.

1st Revision - authors' response 06 February 2014

***Referee #1** (Comments on Novelty/Model System):*

Abnormal vascularization of the retina for those with wet macular degeneration, diabetic retinopathy and retinopathy of prematurity.

There are 2 agents commonly used for retinopathy- VEGF antibody and VEGF Trap made as a recombinant protein from domains of VEGFR-1 and VEGFR-2. The problems with these is that they may leak systemically and have effects in premature babies which can be detrimental. Also they may not localize specifically to abnormal angiogenesis areas in the retina and their short duration of effect requiring injections every month or at best 2 months in adults.

The investigators generated a novel form of VEGF receptor, VEGF Trap, which had sticky domains coding exon 6, 7 and 8 or variants of those of VEGF. These domains of VEGF are responsible for its binding to heparin in extracellular matrix. They used appropriate controls with and without sticky traps as well as the initial VEGF Trap molecule without any of these domains, as clinically used.

The initial test was in tumour angiogenesis with cell lines expressing the various types of traps. This led to the selection of sticky trap 6, 7, 8 and its recombinant expression. Systemic pharmacology was undertaken to show that it was rapidly metabolized, did not localize in normal tissues and did not interfere with wound healing. However, systemic injection could block tumour angiogenesis.

It showed minimum toxicity in normal tissues compared to VEGF Trap, particularly investigating function in neonates and on tracheal blood vessels. It then undertook studies with intraocular injection into the vitreous. This showed the sticky trap has much better retention for at least 12 days compared to VEGF Trap, which had disappeared by 2 days.

Sub retinal injections were also given. This again showed retention in the sub retinal space. In comparative studies of the oxygen induced retinopathy the sticky trap was superior in all endpoints and showed no systemic leak. Additionally, it was not toxic to the eye in terms of normal retinal function. Thus, this represents a unique new molecule with exceptional pharmacology properties for local control of abnormal angiogenesis in the eye. This represents a significant step forward in all aspects of safety and enhanced efficacy and therefore meets a major need for dealing with retinopathy of prematurity and probably also substantial benefit for adult eye disease.

I think the work was extremely well performed with appropriate control, including consideration on the potential value in tumour angiogenesis where selective binding to vasculature could increase the safety margin.

Major comment

The one missing issue here, however, is understanding why it binds so specifically around the tumours and the vascular abnormalities of prematurity but not to normal tissues or to wounding or to other areas which are known to have high VEGF for example the glomerulus- so why is there this highly specific binding? What are the binding components, what is the basis for the selectivity; this is not mentioned in the discussion and there is no experimental data on this.

One might a priori have expected it to bind everywhere that VEGF is detected, but it is clearly not the case. I would recommend at least discussion of this and perhaps some initial evidence for binding differentiated extracellular matrix around tumours or from abnormal ocular vascular could be provided.

Overall we would like to thank the reviewer for the positive comments, for recognizing the unique pharmacological properties of Sticky-trap and its potential use in the clinic for ROP and DR.

We would like to clarify the mechanism of the specific retention of Sticky-traps at the site of delivery.

As the reviewer mentions, in order to achieve strictly local activity of Sticky-trap we took advantage of the heparin binding domain (HBD), which is common to many growth factors (Munoz & Linhardt, 2004). HBD is composed by a series of positively charged amino acids, which interact with the negatively charged sulphate of heparan-sulphate glycosaminoglycans, which leads to binding of the growth factor in the extracellular matrix. VEGF has 3 different HBDs due to alternative splicing, shown in Supplementary Figure 1B. In this study we used the same HBDs of

VEGF in order to create various isoforms of Sticky-trap. Previous studies have shown that VEGF181, which contains the HBD of both exon 6 and 7 does not circulate at all, and remains at the site of secretion (Park et al, 1993). Similarly, in this study we show that Sticky-trap does not leak in the circulation and remains at the site of delivery. The later is strictly due to the nature of the HBD that we used. We would like to clarify that the specificity of the localization is determined by the delivery site; therefore, if Sticky-trap is injected in a different organ it will remain in that organ.

We have included this relevant information in the introduction, as well as in the discussion:

1. Introduction: "The storage of numerous growth factors in the extracellular matrix, as well as the creation of morphogen gradients during development, is controlled by the interaction between the heparin-binding domain (HBD; a sequence of positively charged amino acids) of the growth factor and extracellular-matrix heparan-sulphate proteoglycans (HSPGs). VEGF contains two regions with HBDs coded by exons 6 and 7. Alternative spicing of these exons results in distinct isoforms. VEGF121 is missing both exons and is therefore completely soluble. VEGF145 and VEGF165 contain exon 6 and 7, respectively and are therefore partially soluble. VEGF189 has both exons and is strictly retained at the secretion site (Houck et al, 1992; Park et al, 1993)."

2. Discussion: "The ability of Sticky-traps to remain at the site of delivery was achieved by engineering the original VEGF-trap to contain a strong heparin-binding domain, and thus to be able to bind to negatively changed heparan-sulphate glycosaminoglycans of various HSPGs of the extracellular matrix and cell surface."

Referee #2 *(Comments on Novelty/Model System):*

This is exceptionally thoroughly and convincingly performed study that has obvious clinical potential. I do not have additional requests regarding new experimentation (this has not happened for many years, although I review tens of manuscripts every year).

Referee #2 (Remarks):

This is exceptionally thoroughly and convincingly performed study that has obvious clinical potential.

We would like to take this opportunity to thank the reviewer for the very kind comments, and for recognizing the clinical potential of this novel antiangiogenic biologic.

Referee #3 *(Remarks):*

This manuscript reports a very exciting study of a novel pharmacological inhibitor VEGF named VEGF Sticky-trap. Compared to traditional VEGF-trap, Sticky-trap shows a similar anti-angiogenic effect and strong capacity to inhibit VEGF signalling. Uniquely, Sticky-trap binds to extracellular matrix through heparin-binding domains and remains in the administration site without entering the circulation. The study demonstrates that local administration of Sticky-trap has little impact on distal wound healing, suggesting that this treatment will have low systemic side-effects. Thus, this novel inhibitor of VEGF may provide an effective and safer treatment for ocular neovascularization and tumour.

We would also like to thank the reviewer for recognizing the unique characteristics of Sticky-trap and its potential use as an effective and safer antiangiogenic biologic. We also appreciate hers/his constructive comments, which allowed us to further clarify the different aspects of Sticky-trap in the manuscript.

Main points:

1. Fig. 1G shows that Sticky-traps suppressed endothelial cell proliferation in a dose-dependent manner, while in VEGF-trap and Short-trap groups, the inhibitory effect reached a plateau at doses of 10 pM and higher. Interestingly, results from Fig. 1F and Suppl. Figs. 3 and 4 suggest that VEGF-trap and Short-trap had similar, if not stronger, effects on VEGF and VEGF-R2 inhibition. What potential mechanisms could lead to Sticky-trap's more potent inhibition on VEGF-induced proliferation?

Indeed the reviewer is correct to point out the superior ability of Sticky-trap to inhibit endothelial cell proliferation compared to VEGF-trap and Short-trap. Although at this moment we do not have a clear understanding of this unexpected and positive characteristic of Sticky-trap, we believe that it can be due to the fact that both Sticky-trap and VEGF have the same heparin-binding domain (HBD). Hence, it is possible that Sticky-trap not only irreversible binds VEGF, but also competes with it for binding to: (a) the extracellular matrix, and (b) to neuropilin-1 (Nrp-1). The first may decrease VEGF bioavailability, while the second might compromise the ability of VEGF to activate VEGF-R2, since for this to take place VEGF has to bind to Nrp-1 and it is known that exons 7 and 8 of the HBD contribute to this physical interaction.

We have added the text below in the relevant result section: "The enhanced ability of Sticky-trap to inhibit HUVEC proliferation in doses higher than 10pM is presumably attributed to the fact that both Sticky-trap and VEGF have the same HBD. Therefore, it is possible that Sticky-trap can affect the bioavailability of VEGF by competing to available binding sites in the ECM, as well as compromise the ability of VEGF to bind to Neuropilin-1, which is mediated through exons 7 and 8 of the HBD, and is required for VEGFR-2 activation (Parker et al, 2012)."

2. Suppl. Fig. 18: It seems that Sticky-trap does not across the inner limiting membrane after an intravitreal injection. Thus, it is understandable that intravitreal injection of Sticky-trap will not affect retinal function and outer retina integrity (Fig. 9). But would retinal ganglion cells and neurons of the inner retina be affected? Chronic retinal neuronal injury may occur after an injection.

We fully understand the concerns of the reviewer regarding possible effects of Sticky-trap in the ganglion and inner retina cells layers, since it binds in close proximity to this area. Our ERG data indicate that this was not the case, since after prolonged treatment of 6-weeks we did not observe any decrease in the amplitude of the b-wave during the scotopic ERG, which would indicate defects in the ganglion cells or other cell types of the inner nuclear layer (Figure 9D).

We have further discussed this in the manuscript in the relevant result section: "Using both scotopic and photopic ERGs we demonstrated that the function of rod (a-wave; scotopic ERG, and flicker; photopic ERG) and cone (flash; photopic ERG) photoreceptors was not affected (**Figure 9B-G**). In addition, the unaltered b-wave amplitude of scotopic ERG indicates that the function of the cells of the inner nuclear layer, such as bipolar and Muller cells, as well as the function of ganglion cells was also not affected (**Figure 9D**)(Bui & Fortune, 2004; Dong & Hare, 2000). Similar results were also observed one week after a single subretinal injection of Sticky-trap (**Supplementary Figure 19**)."

3. In Suppl. Fig. 17, large amount of Sticky-trap were detected in the inner and outer segment of photoreceptors after a subretinal injection. In this case, would Stick-trap cause any adverse effect on photoreceptors and choroidal blood vessels? Would subretinal injection of Stick-trap be sufficient to suppress choroidal neovascularization, a hallmark pathological feature of wet AMD?

The excess amount of Sticky-trap shown in the inner and outer segment of photoreceptors is the result of spill during the injection, which was performed through the retina in order to reach the subretinal area. The purpose of this experiment was to show that Sticky-trap is able to bind to the subretinal space, and not to propose this as a therapeutic approach in the case of AMD since repeated subretinal injections are not feasible in humans. Nevertheless, we also examined whether a single subretinal injection of Sticky-trap had any toxicity, and as we show in Supplementary Figure 19 this was not the case since ERG studies were normal.

As we mentioned in the discussion we envision that Sticky-trap can be delivered in the subretinal area using various methods such as slow-release implants, liposomes, viruses, and RPE cells. This is a separate area of investigation and thus we do not feel that it can be included as part of this study.

4. If Sticky-trap does not across the inner limiting membrane and get into the retina, how could the trap bind to VEGF and inhibit VEGF/VEGF-R function in retinal tissue? Biochemical assays of retinal VEGF levels and retinal VEGF-R activation (tyrosine phosphorylation assay) should be performed to confirm the effects of Sticky-trap on retinal VEGF signalling and to delineate whether the anti-angiogenic and anti-permeability effects of Sticky-trap is through inhibition of retinal VEGF system.

As we mentioned above and in the discussion, the focus of this study was to prove the efficacy and safety of recombinant Sticky-trap to inhibit abnormal neovascularization in during DR and ROP, in which new vessels grow along the surface of the retina and advance into the posterior hyaloid face of the eye. In order to access the ability of Sticky-trap to inhibit the formation of the later, we used the relevant mouse model of OIR. The possible applicability of Sticky-trap for inhibition of vessels in the subretinal space and its use for treatment of AMD will be the purpose of another study, using a cell based method for delivery, for example

5. Would the binding of Sticky-trap to ECM affect the pharmacokinetics and anti-VEGF efficacy in the treatment of retinal/choroidal neovascularization? Please discuss.

Previous studies indicated that both the thickness of the inner limiting membrane (ILM), and the amount of heparan-sulphate proteoglycans (HSPGs) in the ILM, increase in patients with DR (Matsunaga et al, 2005). Therefore, the increase of HSPGs will likely allow for an even higher capacity for Sticky-trap binding and enhanced anti-VEGF efficacy. We have now briefly mentioned this point in the discussion of the manuscript.

Discussion section: “Previous studies have shown that both the thickness of the ILM, as well as the amount of HSPGs, increase with disease progression in the case of DR (Hanneken et al, 1991; Matsunaga et al, 2005). The later will allow for even higher retention of Sticky-trap in patients with advanced disease which may result in higher clinical benefit.”

6. In some metastatic tumours, local injection may be difficult to apply. Potential implication of Sticky-trap in the treatment of these diseases should be discussed.

We apologize to the reviewer if we gave the wrong impression that we intend on proposing the use of Sticky-trap for treatment of cancer. As we mentioned in the text (please see below) the use of cancer mouse models was strictly done in order to initially evaluate the antiangiogenic and local effect of Sticky-trap. We do not propose that Sticky-trap can be used in oncology at this point.

Results section: “Although our intention here is to propose the use of Sticky-traps as therapeutic agents to suppress pathological neovascularization in eye diseases, in order to initially explore and evaluate the in vivo effect of Sticky-traps with ease, we used tumour xenograft assays”.

References

Bui BV, Fortune B (2004) Ganglion cell contributions to the rat full-field electroretinogram. *The Journal of physiology* **555:** 153-173

Dong CJ, Hare WA (2000) Contribution to the kinetics and amplitude of the electroretinogram b-wave by third-order retinal neurons in the rabbit retina. *Vision Res* **40:** 579-589

Hanneken A, de Juan E, Jr., Lutty GA, Fox GM, Schiffer S, Hjelmeland LM (1991) Altered distribution of basic fibroblast growth factor in diabetic retinopathy. *Arch Ophthalmol* **109:** 1005-1011

Houck KA, Leung DW, Rowland AM, Winer J, Ferrara N (1992) Dual regulation of vascular endothelial growth factor bioavailability by genetic and proteolytic mechanisms. *J Biol Chem* **267:** 26031-26037

Matsunaga N, Ozeki H, Hirabayashi Y, Shimada S, Ogura Y (2005) Histopathologic evaluation of the internal limiting membrane surgically excised from eyes with diabetic maculopathy. *Retina* **25:** 311-316

Munoz EM, Linhardt RJ (2004) Heparin-binding domains in vascular biology. *Arterioscler Thromb Vac Biol* **24:** 1549-1557

Park JE, Keller GA, Ferrara N (1993) The vascular endothelial growth factor (VEGF) isoforms: differential deposition into the subepithelial extracellular matrix and bioactivity of extracellular matrix-bound VEGF. *Mol Biol Cell* **4:** 1317-1326

Parker MW, Xu P, Li X, Vander Kooi CW (2012) Structural basis for selective vascular endothelial growth factor-A (VEGF-A) binding to neuropilin-1. *J Biol Chem* **287:** 11082-11089

2nd Editorial Decision 24 February 2014

Thank you for the submission of your revised manuscript to EMBO Molecular Medicine. We have now received the enclosed reports from the Reviewers that were asked to re-assess it. As you will see the reviewers are now globally supportive and I am pleased to inform you that I am prepared to accept your manuscript for publication pending the following final amendments and requests:

1) We note that the blots in Fig. 1D especially the "Sticky-trap" strip, appear too contrasted and too narrowly cut. Please provide a better image and the source data for this Figure.

2) As per our Author Guidelines, the description of all reported data that includes statistical testing must state the name of the statistical test used to generate error bars and P values, the number (n) of independent experiments underlying each data point (not replicate measures of one sample), and the actual P value for each test (not merely 'significant' or 'P < 0.05').

3) We would need a short list (up to 5) of bullet points that summarize the key NEW findings. The bullet points should be designed to be complementary to the abstract and will be used online in our new web platform. Please submit this as a separate file.

4) We are now encouraging the publication of source data, particularly for electrophoretic gels and blots, with the aim of making primary data more accessible and transparent to the reader. Would you be willing to provide a PDF file per figure that contains the original, uncropped and unprocessed scans of all or at least the key gels used in the manuscript? The PDF files should be labeled with the appropriate figure/panel number, and should have molecular weight markers; further annotation may be useful but is not essential. The PDF files will be published online with the article as supplementary "Source Data" files. If you have any questions regarding this just contact me.

I look forward to reading a new revised version of your manuscript as soon as possible.

***** Reviewer's comments *****

Referee #1 (Remarks):

All questions have been answered

Referee #3 (Remarks):

The authors have done wonderful work in addressing my questions. The manuscript is suitable for publications.

2nd Revision - authors' response 02 March 2014

We are delighted that you are prepared to accept our manuscript entitled “Local acting Sticky-trap inhibits VEGF dependent pathological angiogenesis in the eye” for publication in EMBO Molecular Medicine.

As per you suggestion we have replaced the image in Figure 1D, and also provided the source data. In addition we have provided the source data for Figure 1B, and Suppl. Figure 3C. Furthermore, we made sure that we have reported the statistical test, along with number of independent experiments and actual P values for each test. A separate file with a short list of bullet points summarizing the key new findings has also been submitted.

I look forward hearing from you, and for our manuscript to be accepted for publication in EMBO Molecular Medicine.
