# Supplementary figures and images for: Local acting Sticky-trap inhibits vascular endothelial growth factor dependent pathological angiogenesis in the eye
Source: EMBO Mol Med. 2014 Apr 4;6(5):604–23. doi: 10.1002/emmm.201303708 (PMC4023884; doi:10.1002/emmm.201303708)

Source file for Figure 1B

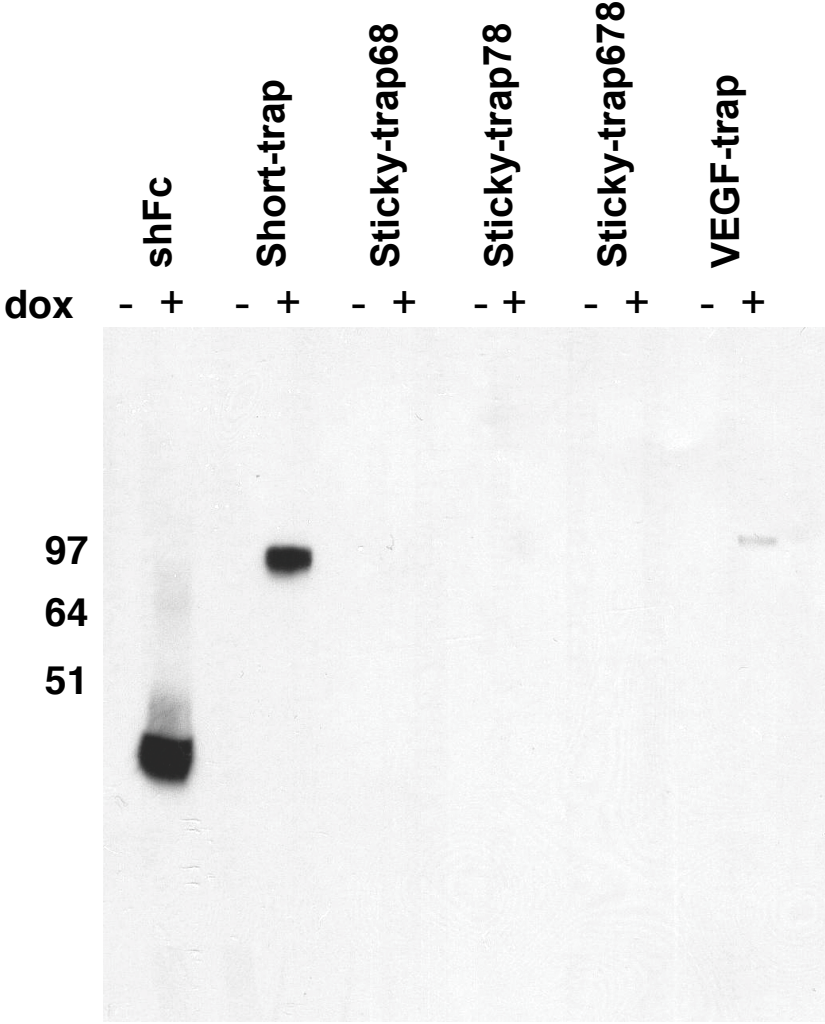

5 seconds exposure time

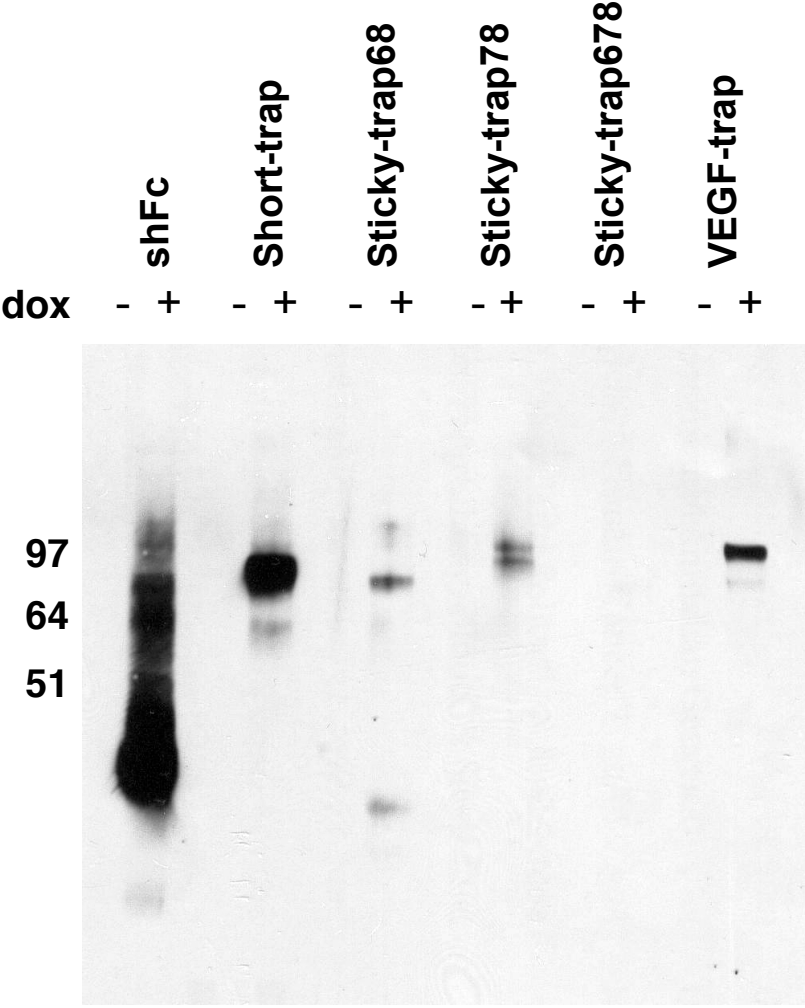

60 seconds exposure time

Source file for Figure 1D

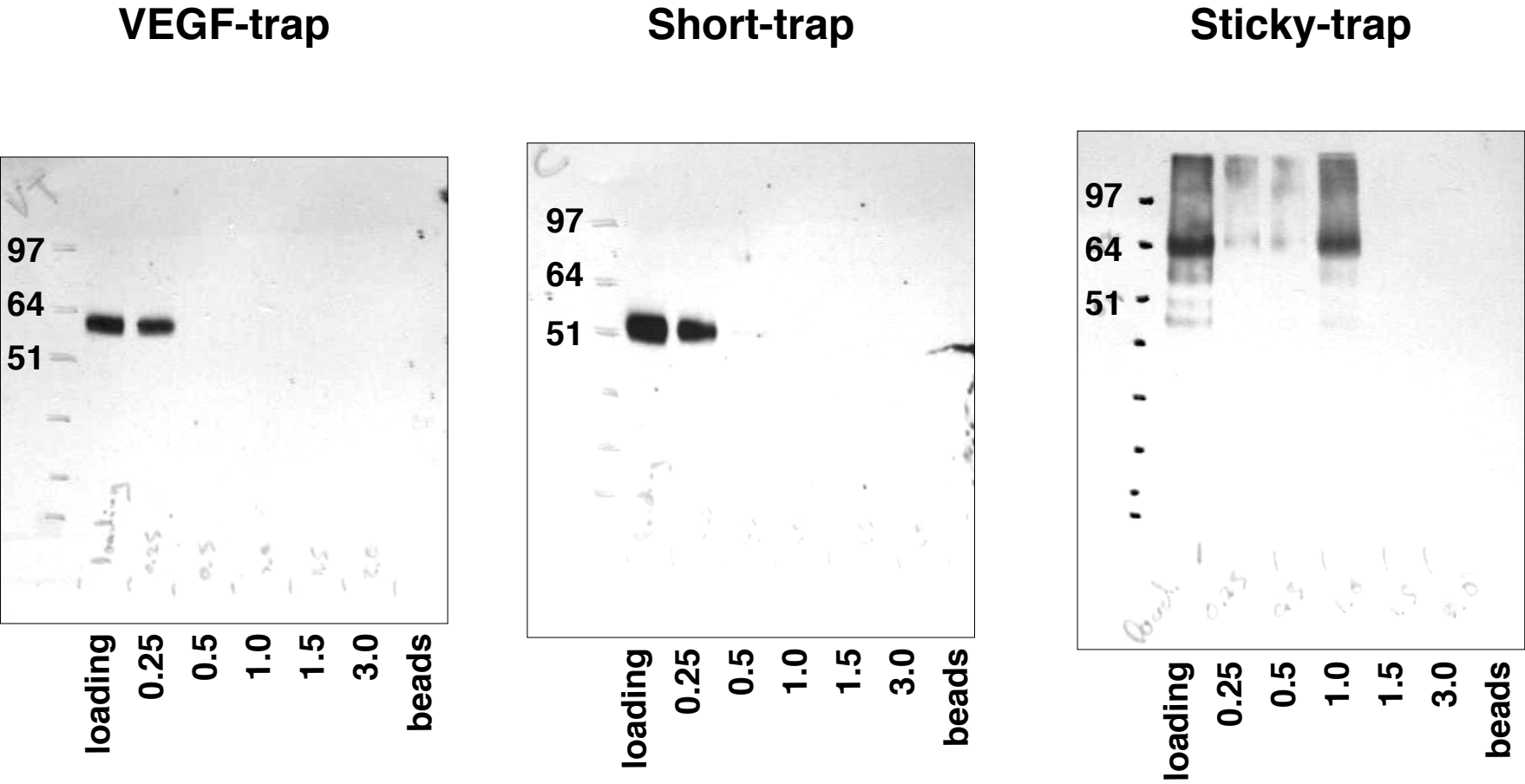

Supplement: Supplementary file 22 [file emmm0006-0604-sd22.pdf]

Source file for Suppl. Figure 3C

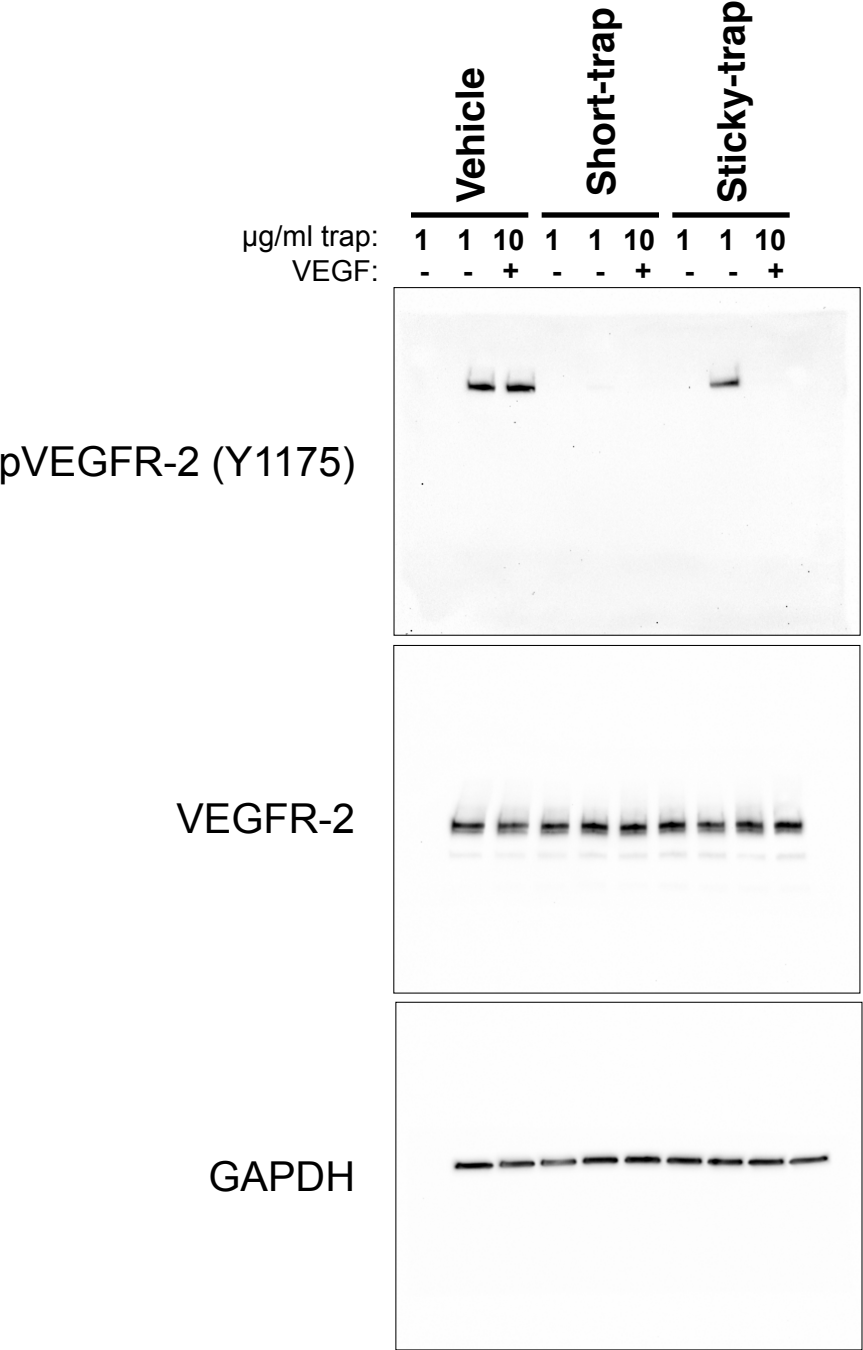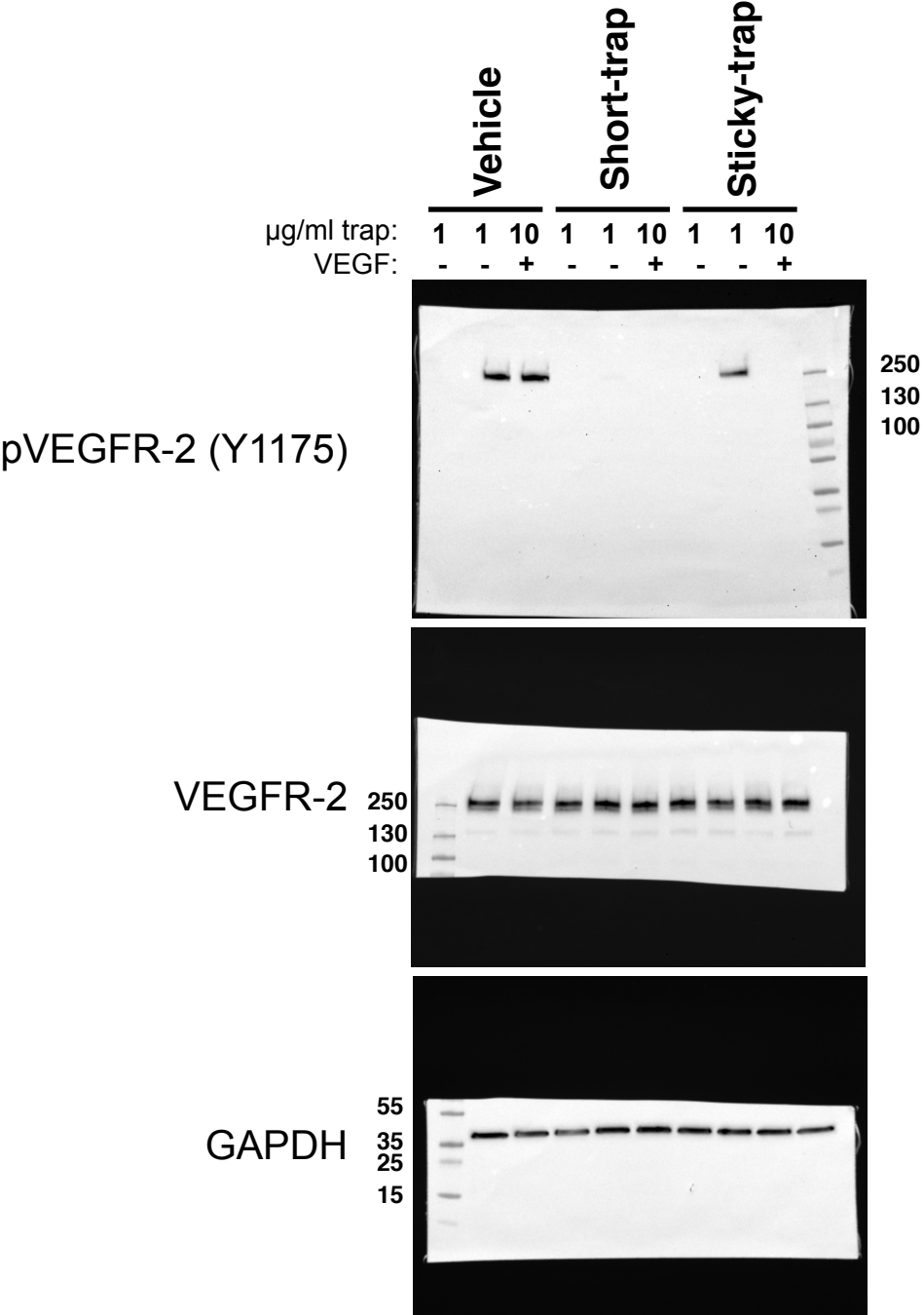

Supplement: Supplementary file 23 [file emmm0006-0604-sd23.pdf]
